# An Overview of Literature Topics Related to Current Concepts, Methods, Tools, and Applications for Cumulative Risk Assessment (2007–2016)

**DOI:** 10.3390/ijerph14040389

**Published:** 2017-04-07

**Authors:** Mary A. Fox, L. Elizabeth Brewer, Lawrence Martin

**Affiliations:** 1 Department of Health Policy and Management, Bloomberg School of Public Health, Johns Hopkins University, Baltimore, MD 21205, USA; mfox9@jhu.edu; 2 Office of the Science Advisor, U.S. Environmental Protection Agency, Oak Ridge Institute for Science and Education (ORISE), Washington, DC 20004, USA; brewer.beth@epa.gov; 3 Office of the Science Advisor, U.S. Environmental Protection Agency, Washington, DC 20004, USA

**Keywords:** cumulative risk assessment, nonchemical stressor, chemical mixture, vulnerable populations, community health, environmental justice, ecological health

## Abstract

Cumulative risk assessments (CRAs) address combined risks from exposures to multiple chemical and nonchemical stressors and may focus on vulnerable communities or populations. Significant contributions have been made to the development of concepts, methods, and applications for CRA over the past decade. Work in both human health and ecological cumulative risk has advanced in two different contexts. The first context is the effects of chemical mixtures that share common modes of action, or that cause common adverse outcomes. In this context two primary models are used for predicting mixture effects, dose addition or response addition. The second context is evaluating the combined effects of chemical and nonchemical (e.g., radiation, biological, nutritional, economic, psychological, habitat alteration, land-use change, global climate change, and natural disasters) stressors. CRA can be adapted to address risk in many contexts, and this adaptability is reflected in the range in disciplinary perspectives in the published literature. This article presents the results of a literature search and discusses a range of selected work with the intention to give a broad overview of relevant topics and provide a starting point for researchers interested in CRA applications.

## 1. Introduction and Background

### 1.1. Objectives of This Manuscript

This paper is organized and written in a manner that is intended to function as a reference guide for overarching concepts, commonly used techniques, and innovative methods for cumulative risk assessment (CRA). The human health, ecological health, and ecosystem services results sections introduce and briefly highlight papers that are presented in the tables in the Supplementary Materials. This manuscript presents work from each of these subject areas in an effort to address recommendations to develop more integrative approaches to assessing cumulative risks. Although the focus is on human health, papers from the ecological literature provide complementary or potentially adaptable methods and approaches. The discussion section presents a synthesis of these papers. 

Some sections further highlight certain articles out of those presented in the tables. It is not the intent of the authors to comment on data quality or to provide a ranking of the presented studies, but to direct attention to papers that represent a certain subject or that present essential concepts and methods. There are numerous studies from many different disciplines that could provide useful methods to CRA, but because CRAs can be stressor-oriented, effects-based, or community or population specific, it is impossible to predict what studies might be relevant. Therefore, papers are highlighted because they focus on providing information that is particularly important to developing new and innovative methods for CRA, or have broad applicability to many aspects of CRA. Because CRA can be designed to focus analysis on a diverse range of issues, and draws from a wide range of information, the selection of literature in this review is intended to provide a perspective and starting point for interested researchers.

### 1.2. What Is Cumulative Risk Assessment (CRA)?

In guidance documents for CRA [1,2], the U.S. Environmental Protection Agency (EPA or the Agency) defines a CRA as an analysis, characterization and possible quantification of the combined risks to health or the environment from multiple agents or stressors. The National Research Council’s (NRC) Committee on Improving Risk Analysis Approaches Used by the EPA proposed CRA be defined as evaluating an array of stressors (chemical and nonchemical) to characterize quantitatively―to the extent possible―human health or ecological effects, taking account of such factors as vulnerability and background exposures [3]. The concept of cumulative effects has roots in the Council on Environmental Quality (CEQ) regulations implementing the National Environmental Policy Act (NEPA) of 1969 (Pub. L. No. 91-190), which included some principles of cumulative effects analysis for environmental impact assessments under NEPA [4]. Some of the key developments in cumulative risk concepts over time are provided in Supplementary Materials (Table S1). 

### 1.3. Literature Selection and Synthesis Approach

In developing this report, PubMed, Web of Science, and Scopus were comprehensively searched on two occasions; first in 2011, and again in 2016 to update the original document. The databases were searched for the terms “CRA”, “integrated assessment”, “psychosocial stress”, “chemical mixtures”, and “ecological risk” from January 2007 to October 2011. They were searched again in May 2016 for the same terms listed above with the addition of the terms “disproportionate risk” and “ecosystem services”. Search terms were selected based on consultation with EPA Risk Assessment Forum staff. 

Search terms were chosen to explore a defined set of topics based on consultation with EPA staff. For this reason, a systematic review approach for this inquiry was not employed. Literature selection methods were instead modified to examine identified topics, and to capture as much relevant research as possible. Traditional methods for formulating systematic review questions would be more applicable for a CRA if tailored for a specific population, set of stressors, and health outcome. The type of information that could potentially be useful for future CRAs in specific populations or among multiple stressors is difficult to predict and should be tailored to fit a prescribed purpose. The search terms selected for this review are indicative of the evolving nature of CRA (e.g., the search term “ecosystem services” was added in the update to the original review). 

The selected literature is presented in three sections—human health, ecological health, and ecosystem services. The ecosystem services section presents literature that bridges the gap between human and ecological health. The papers reviewed and presented in the ecosystem services section were published between 2011 and 2016, as this was a new term added later in the review.

Included in the references are two relevant studies [5,6] submitted through a Federal Register notice requesting information and citations on methods for CRA (Docket ID No. EPA-HQ-ORD-2013-0292). The combined database search resulted in 1752 references from early 2007 through mid-2016.

The articles cited in this review were filtered from the 1752 references. Article titles were reviewed to identify an underlying order to topics in the reference list. Papers are organized according to these topic areas. Questions were designed to capture relevant articles using NRC and EPA-developed CRA definitions and concepts (see Section 1.2) [2,3]. Abstracts were reviewed and filtered using these selection questions:

- Does the article specifically address cumulative risk assessment?
- Does the article specifically address cumulative risk, cumulative effects, combined exposures, multiple routes of exposure, additivity, synergistic or antagonistic effects?
- Does the article address community health or environmental justice?
- Does the article roughly fit into a risk assessment step (Problem Formulation, Dose Response, Exposure Assessment, Hazard Identification, Risk Analysis, Risk Characterization) or a combination of steps?
- Is the article representative of a discipline (e.g., social epidemiology, ecotoxicology) and does it also address its method’s applicability to cumulative or multiple risks, exposures or effects?
- Does the article address common difficulties in evaluating cumulative risks (e.g., approaches for estimating joint action in chemical mixtures, measuring allostatic load (AL), impacts of nonchemical stressors)?
- Does the article address susceptibility or vulnerability?
- Does the article present novel methods for incorporating nonchemical stressors with the evaluation of chemical risks?
- Does the article address any of the recommendations of the NRC [3,7] reports?

**Figure 1:**
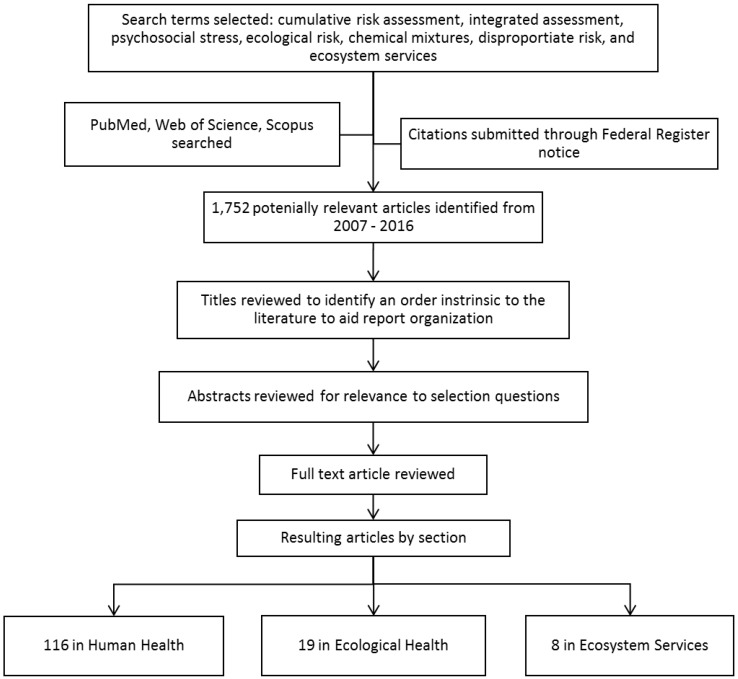
Flow chart of literature selection process.

The resulting full text articles were then examined for inclusion in this review (see Figure 1 for process overview). Other papers cited in this review are for supplying necessary background information or for further discussion.

## 2. Results—Human Health

Key features of cumulative risk and human health papers presented in the tables in the Supplementary Materials and highlighted in this section are summarized in Table 1 below. 

### 2.1. Review Articles 

#### 2.1.1. Nonchemical Stressors and Cumulative Risk Assessment: An Overview

A review by Lewis et al. [8] provides an overview of important CRA concepts and summarizes investigations of the impact of social stressors on health. The investigations are drawn primarily from the air pollution epidemiology literature, although a few studies of blood and bone lead are included. The epidemiological research Lewis et al. gathered addressed social stressors as effect modifiers in the relationships between air pollutant exposures and health effects ranging from asthma to mortality. In the subset of studies of blood and bone lead, the outcomes were neurological, including IQ decrement, mental development scores and other cognitive measures. The underlying studies were not re-evaluated for this review, and specific aspects of study design and implementation or variations in how stressors are defined may explain some of the heterogeneity in the findings as reported by Lewis et al. [8]. Despite concluding that the evidence they reviewed offered mixed results regarding the impact of nonchemical stressors, Lewis and co-authors were optimistic about the potential to apply epidemiological research methods to quantify the effects of nonchemical stressors. Recommendations offered by Lewis et al. for advancing CRA included a few topics that apply to risk assessment in general, such as better quantification of exposure and focused efforts to apply epidemiological findings in risk assessments. The other critical needs for cumulative risk research and practice identified were:

- Continued attention to extrapolation from animal studies (i.e., How well does stress induced in animals relate to psychosocial stress in humans?).
- Identification of biomarkers that may provide a means to integrate effects of chemical and nonchemical stressors (see [9]).
- Measures or metrics of nonchemical stressors that facilitate dose–response assessment.
- Methods to quantify interactions between chemical and nonchemical stressors and describe differences in dose–response curves.
- Epidemiological evaluations that explore the relative contributions of chemical and nonchemical stressors and how such findings relate to dose–response.
- Methods and metrics for cumulative risk characterization.

In the context of this literature review, Lewis et al. made a unique contribution by keying their analysis to the risk assessment “steps” (e.g., hazard identification, exposure assessment). As a caution, however, the review is not systematic and is not intended to be “an exhaustive analysis” [8] (p. 2022). Another issue to note, observed by Lewis et al. [8] and in the many studies reviewed below, is that there are many ways to represent or measure social stress, ranging from area-level social stress indicators developed from census information to individual self-reports and survey instruments, indicators of social class, and a neighborhood-level psychosocial hazard index.

#### 2.1.2. An Update on Phthalates Toxicology

In their update, Rider et al. [10] documented then-recent work on phthalates and other anti-androgens providing an update on the science reviewed by the NRC Committee on the Health Effects of Phthalates [7]. Rider et al. reviewed findings of studies that evaluated various types of mixtures of male reproductive toxicants. The various mixtures tested represented different mechanisms of toxicity (e.g., androgen receptor agonist, enzyme inhibition) as well as diverse toxicity pathways (androgen- vs. aryl hydrocarbon-receptor signaling). The effects observed in these studies were well-predicted by dose-additivity and exceeded predictions made on the basis of response-additivity. The authors concluded:

“… our results indicate that compounds that act by disparate mechanisms of toxicity to disrupt the dynamic interactions among the interconnected signaling pathways in differentiating tissues produce cumulative dose-additive effects, regardless of the mechanism or mode of action of the individual mixture component.”

[10] (p. 443)

Rider et al. [10] present evidence from their studies of phthalates and other anti-androgens and argue for expanding the organizing principle for CRAs from “common mechanism of toxicity” to include “common adverse outcome” as well, as recommended by the NRC Committee on the Health Effects of Phthalates [7]. 

### 2.2. Conceptual Developments 

Types of papers categorized as conceptual developments in CRA are summarized in Table 1 with additional details in Table S2. These include thought pieces on cumulative risk from different disciplinary perspectives, e.g., anthropology [11], sociology [12], and health geography [13]; frameworks and approaches for conducting or designing CRAs [5,14,15,16,17,18,19,20], biomonitoring approaches [9], and epidemiological approaches [21]; and definitions and discussion of important concepts, including interindividual variability [22], AL [23], and psychosocial stressors [24]. 

### 2.3. Cumulative Risk Methods and Applications for Human Health

The types of methods and applications of cumulative risk represented in the literature review fall into four main categories: geographic information systems (GIS) (see Table S3); emerging work in biomarkers, genetics and “omics” (see Table S4); varied modeling approaches (see Table S5); and continued application and some refinements of CRA for pesticides. The latter work is summarized in the following Section 2.3.1 (the papers are not included in the tables). 

#### 2.3.1. Applications and Developments in Cumulative Exposure and Risk Assessment of Chemical Mixtures

##### Pesticides

Methods used by EPA’s Office of Pesticide Programs have been adopted by others to evaluate exposure and risk resulting from exposure to pesticides with a common mechanism of action and their residues in diet or particular foods [25,26,27]. Bosgra et al. [28] reported on development of an isobologram approach to predict effects from chemical mixtures, but later reported a preference for a biological/physiological model [29,30,31]. Muller et al. [32] used relative potency factors to evaluate exposures and risks from anti-androgenic pesticides in food. Abdo et al. [33] used an in vitro model to evaluate hazard, mode of action, and population variability in response to pesticide mixtures. Jensen et al. [34] used the hazard index approach to assess chronic cumulative dietary exposure to pesticide mixtures. Kennedy et al. [35] demonstrated probabilistic approaches for uncertainty analysis for pesticides, including a discussion of probability from Bayesian and frequentist perspectives. Moretto [36] recommended in vitro testing and PBPK modeling to refine pesticide groupings. 

The European Food Safety Authority (EFSA) released a scientific opinion on assessing the cumulative risk of pesticide mixtures in food in support of the “common adverse outcome” approach recommended in the NRC’s phthalates report [7]. The opinion recommended dose addition (DA) as a conservative default method for the assessment of pesticides with a dissimilar mode of action (MOA), given that they produce common adverse outcomes in the same target organs or systems [6]. 

##### Other Chemical Mixtures

Dewalque et al. [37], Hartmann et al. [38], and Wang et al. [39] provided examples of current CRAs of phthalate exposure in geographically distinct populations; Dewalque et al. [37] and Hartmann et al. [38] addressed phthalate mixtures in the Belgian and Austrian general populations respectively, while Wang et al. [39] focused on children in three areas in China, finding that children living in manufacturing intensive areas are at a highest risk of phthalate exposure. Pelallo-Martinez et al. [40], also focusing on children in industrial areas, used urinary biomarkers to assess exposure to a mixture of lead, benzene, toluene, and polyaromatic hydrocarbons (PAHs), and find that variation in the levels of PAHs in the mixture modifies the genotoxic and hematological effects of exposure. Maffini and Neltner [41] explored deficits in traditional risk assessment methods, identified over 300 food additives that may cause adverse effects in the developing brain, and urged more focus on cumulative biological effects. Henn et al. [42] focused on children’s health outcomes in a review of recent epidemiological literature examining chemical mixtures.

The following papers explored various aspects of exposure to chemical mixtures and demonstrated crossover in techniques among disciplines, conceptual advances, and new assessment methods:

- Lee and Jacobs [43] discussed glutathione depletion and mitochondrial dysfunction resulting from chronic exposure to persistent organic pollutants (POPs), and how physical stressors and behavioral factors can be counteractive measures mitigating the impacts of POP exposure.
- Orton et al. [44] demonstrated combination in vitro effects of mixtures containing a large variety of current use androgen receptor antagonistic chemicals.
- Ge et al. [45] characterized the impacts of exposure to metal mixtures in vitro using a systems biology approach integrating proteomics, bioinformatics, statistics, and computational toxicology.
- Hadrup [46] suggested that chemicals should be prioritized based on potency and risk of exposure, and an overall estimate of chemical mixture effects on all targets would be a more effective strategy for risk assessment than dose addition and grouping by target organ.

Three scientific committees of the European Commission’s Directorate General for Health and Consumers published recommendations on the evaluation of chemical mixtures in 2011, “Toxicity and Assessment of Chemical Mixtures”. The report recommended DA when MOAs are similar and independent action (IA) when chemicals act through dissimilar modes of action. DA is also recommended as a default method in the absence of MOA information. The report’s discussion and recommendations include consideration of mixture evaluation in both human and ecological assessment. 

#### 2.3.2. Highlights from the Geographic Information Systems Literature

Studies utilizing GIS and spatial analysis contribute to epidemiologic evidence for geographic influences on multistressor exposures and health outcomes. In Table S3, papers to note in the literature on GIS are Basara and Yuan [47] and Briggs et al. [48]. Basara and Yuan [47] described a GIS that combines social, physical/chemical and health outcome data sets; it is an example of a data system developed from surveillance data. The work of Briggs et al. [48] takes the next step and employs a GIS database that evaluated environmental inequities in England. In addition, Salinas et al. [49] and Huang and London [50] developed methods to assess the impact of multiple environmental stressors on community health using spatial analysis. Although listed among the important contributions to the Vulnerable Populations literature in Table S6, the work of Alexeeff et al. [51] and McDonald et al. [52] should also be noted as a significant addition to the GIS literature. The work of Shmool et al. [53] looked at psychosocial stressors and air pollution in New York City using GIS-based analysis.

#### 2.3.3. Highlights from the Biomarker, Genetic and “Omics” Literature 

Several interesting papers were found in the emerging area of biological methods for cumulative risk, and are presented in Table S3. Of note are the biomarkers examples [54,55,56,57] from human studies and the toxicological work of Hendriksen et al. [58]. Two papers were found that discussed the “how-to” of gene-environment studies and epigenetics that will be informative for designing cumulative risk studies using these approaches [59,60]. Innovative papers from Daughton [61] and Gennings et al. [62] explored the potential of biomarker research from a systems biology perspective. Bonefeld-Jorgensen et al. [63] found ex vivo integrated biomarkers of POP mixtures have the potential to clarify pathways from emissions to health risks. Mentioned in Conceptual Developments Section 2.2 above, Smith et al. [20] described how the exposome can be used to assess cumulative risks, and take steps forward in characterizing a variety of current research efforts, including the “Public Health Exposome” [64], that have potential to advance CRA. 

Although CRA studies with biomonitoring data continue to develop, this area is challenging. There are many scientific and technical considerations such as:

- Timing of exposure and sampling—it may be difficult to obtain samples of compounds with short half-lives in the body.
- Difficulty in determining the source, pathway, or duration of exposure from biomarkers.
- Characterization of biomarkers. It is unclear how well-characterized the biomarkers are with respect to their links to exposure, susceptibility or health effects [9].

Despite these challenges, Smith et al. [20] demonstrated that exposomic analysis has contributed to advancements in the characterization of biomarkers, and elucidation of biological responses at the individual and population level to chemical and nonchemical stressors originating in different types of environments (i.e., natural, built, social, and political). 

#### 2.3.4. Measures and Models 

The papers categorized as relating to “measures and models” are summarized in Table 1 with additional detail in Table S5. Contributions to measures and models for CRA include inventories of publicly available measurement instruments and databases [65,66,67]; development of indices of social and psychosocial factors [68,69]; studies of AL [70,71]; an animal model of chronic social stress [72]; evidence synthesis methods for epidemiological data [73]; and dose–response studies of chemical and non-chemical stressors [74,75,76]. 

### 2.4. Cumulative Risk Examples—Vulnerable Populations

Schwartz et al. [77] explored sources of vulnerability and susceptibility including age, socioeconomic position, and psychosocial stress, and provide empirical examples of how these factors modify risk from exposure to lead and air pollution. Table S6 provides a summary of studies of vulnerability in key populations and lifestages of concern including children and environmental justice communities. Theall et al. [78] and Dulin-Keita et al. [79] evaluated adverse physiological responses to neighborhood-level stressors in children. Other examples of children’s cumulative risk are several studies of air pollutants, stress and childhood asthma or lung function; Pearlman [80] reviewed this topic area. 

Among the studies of air pollutants, stress and childhood asthma or lung function presented in Table S6, three reports show that presence of home-related stress increases vulnerability to exacerbation of asthma or reduced lung function [81,82,83]. The work of Hoffmann et al. [84] reported complex results, finding that although socially disadvantaged children experienced higher exposures to total suspended particulates (TSP) and tobacco smoke and had more unfavorable living conditions, they were less likely to report respiratory diseases but more likely to have abnormal lung function in clinical testing. Explanations offered for the complex findings were selection or reporting bias and biologic interactions [84]. Additional results on childhood asthma and environmental exposures from the Asthma Coalition on Community, Environment, and Social Stress Project will be forthcoming based on the work of Wright et al. [85]. Erickson and Arbour [86] reviewed studies examining socioeconomic risk factors as effect modifiers (rather than confounders) of the effect of exposure to air pollution on pregnancy outcomes, suggesting that socioeconomic stressors and air pollutants share similar etiologic pathways and recommending targeted intervention strategies at multiple levels of organization.

Examples of analysis that examined environmental justice communities include one near a Florida Superfund site [87] and the work of Wing and colleagues who evaluated the health impacts on neighbors of industrial hog farms in North Carolina. They are exemplary for the design, implementation, and outcomes of community-based participatory research [88,89,90,91]. 

Alexeeff et al. [51] developed and demonstrated a screening methodology for identifying potential environmental justice communities. Further characterizing relationships under the environmental justice framework, Schule and Bolte [92] systematically reviewed epidemiological studies of neighborhood socioeconomic position and objective measures of the built environment on individual health outcomes using multilevel models. 

It should be noted that clear cut definitions of vulnerability and susceptibility are not part of the studies in this review and therefore the authors do not attempt to define the terms and may use the terms interchangeably.

## 3. Results—Ecological Health

Key features of cumulative risk, ecological health, and ecosystem services papers presented in the tables in the Supplementary Materials and highlighted in this section are summarized in Table 2 below.

### 3.1. Conceptual Developments 

The field of ecological risk assessment has a strong experimental component, in contrast to human cumulative risk, which must rely on observational studies or toxicological research that require extrapolation between species. Papers representing conceptual developments in ecological CRA are presented in Table S7. Holmstrup et al. [93] summarized some of the large body of work on ecological risks of combinations of chemical and nonchemical stressors (although in somewhat simple combinations). An important concept that has not been studied extensively is the sequence and timing of exposures [93,94,95,96]. Holmstrup et al. [93] pointed to the need to prioritize the types of stressor combinations likely to be most potent to maximize gains from future research. Lokke [94] summarized some of the highlights of the NoMiracle project that were reported in a special issue of Science of the Total Environment, including databases and experimental test systems. Among conclusions of the NoMiracle work is a need to focus on the receptor rather than particular stressors, echoing the calls for effects-based assessments for human cumulative risk [7,14]. In a discussion of climate change in ecotoxicology, Moe et al. [96] highlighted the importance of studying the effects of current and future climate stress on population vulnerability to toxicants. 

### 3.2. Methods and Applications

Developments in methods for ecological risk (see Table S8) include applications of “omics” (e.g., [97]), fuzzy set theory for dose-response [98] and work on the most efficient experimental designs for mixture research [99]. Al-Salhi et al. [100], Garcia-Reyero et al. [101], and Baylay et al. [102] demonstrated more “omics” applications. 

One theme in ecological health literature was the continued exploration of concentration addition versus independent action as the most appropriate models for assessing joint effects; this topic is addressed separately in Section 3.2.1. 

#### Testing Models of Combined Effects—Continuing Exploration of Concentration Addition (CA) or Independent Action (IA) to Predict Joint Effects

Several papers captured the continuing debate about the most appropriate use of the CA or IA models in predicting effects of mixtures of stressors in ecological populations. Coors and De Meester [103] found that IA was useful in a study looking at predation threat, parasitism and pesticide exposure in *Daphnia magna*. Ferreira et al. [104] reported limitations of the models (i.e., circumstances in which one or both models failed to accurately predict effects of multiple stressors). Ferreira et al. [104] also suggests the need for data on toxicological mechanisms to further advance understanding of the effects of complex mixtures. The work of Cedergreen et al. [105] is summarized in more detail below because it reports on the evaluation of a large database of ecotoxicological studies. This evaluation echoed the findings of other authors but went further to offer general guidance on applying CA and IA for complex multi-stressor assessments.

Cedergreen et al. [105] investigated the argument that IA is the most theoretically correct reference model for predicting joint effects of chemical mixtures with different molecular targets (i.e., MOAs). Their study employed 158 existing data sets representing 98 different mixtures, mostly of pesticides and drugs, in one or more of seven common ecotoxicological test systems. The analysis showed that only 20 percent of the mixtures were adequately predicted by IA and only 10 percent by CA. Approximately 50 percent of mixtures could not be described correctly by either model. Although they could not recommend either model on the basis of accuracy, the authors made three recommendations based on the findings and the populations assessed in the data sets examined:

- CA was recommended as a conservative or protective approach in cumulative assessments for individuals.
- IA was recommended as quantitatively most conservative in assessments addressing multiple species or ecosystem level assessments.
- The selection of the model for a joint effects assessment should consider the purpose and context of the assessment and not just the MOAs for the chemicals of concern.

After evaluating the utility of CA and IA alongside species sensitivity distribution curves for assessing chemical mixture risks in ecosystems, Gregorio et al. [106] concluded that CA can lead to underestimations and IA can lead to under- or overestimations of mixture effects. In a review of pesticide mixtures in aquatic systems, CA is found to be a reasonably accurate and conservative approach for effect estimation [107]. This finding is in agreement with Cedergreen and coworkers’ [105] first recommendation listed above. Using mathematical models, Kamo and Yokomizo [108] investigated effects in chemical mixtures and found CA to be accurate only at low concentrations. At higher concentrations, mixture effects can be predicted by CA when MOAs are exactly the same, whereas mixtures characterized by similarities in the MOAs generally produce nonlinear effects.

Backhaus and Faust [109] proposed a tiered approach for mixtures risk assessment, where applying CA is suggested as a conservative and precautious first-tier approach regardless of the MOAs in the mixture. Consideration of IA in the second tier happens only if the calculated risk quotient in the first tier indicates potential risk, or if CA produces an overestimation of risk based on expert opinion. MOA analysis is a last resort if there are considerable differences in effect estimates derived from CA and IA. 

A review of mixture toxicity assessments from the last 20 years from Altenburger et al. [110] pointed out that recent mixture studies employing CA or IA are still ambiguous. To reduce ambiguity, the authors suggest more researchers study the utility of CA and IA extended to the molecular level using transcriptomic, proteomic, and metabolomics approaches. Studying CA and IA at the molecular level will aid in better understanding of MOAs, provide better information for interspecies extrapolations, and improve extrapolations from short-term studies to long-term exposure scenarios [110]. 

### 3.3. Cumulative Risk Examples—Ecological Health

Langmead et al. [111] reported a cumulative risk study of the influence of country-level societal decision making on ecological resources with a case study of the Black Sea region. The framework for the study was the Drivers-Pressures-States-Impact-Response (DPSIR) conceptual model [111]. This work seems to be a unique contribution in terms of its scope and approach in characterizing impacts of very large social development processes and in the use of Bayesian belief networks as a tool for combining information. Landis et al. [112] also used a Bayesian network relative risk model to assess the effects of mercury contamination along with chemical and physical stressors on multiple endpoints in a Virginia river. They suggest that risk management plans considering all stressors are more effective than plans focusing on regulatory criteria for a single chemical (mercury). The study also demonstrates the Bayesian network as an effective tool for adaptive management plans, capable of updating risk measures based on proposed interventions. 

The ecotoxicology literature has many examples of chemical and physical stressor combinations, such as pesticide and temperature change or low oxygen conditions [93,113]. Of note in Table S9 are studies reflecting a current trend of exploring the joint effects of climate change and toxicant exposure [114,115]. The work of Vidau et al. [116] provides an example of a chemical and biological stressor combination. 

## 4. Results—Ecosystem Services

### 4.1. Background

The Millennium Ecosystem Assessment (MEA) [117] evaluated links between ecosystem degradation and human well-being and prompted efforts to develop conceptual frameworks for and quantify the effects of ecosystem services on human health and well-being. Because the health and extent of the natural environment affects the quality and abundance of ecosystem services, and human health is affected by the products of natural systems, ecosystem services constitute a causal link between ecological and human health risk assessment. As CRA seeks to evaluate combined risks to multiple stressors, with community health being a major driving force behind its development, enhancing the flow of information between two traditionally disparate disciplines is vital to fully understanding cumulative risks in defined populations. Therefore, this section highlights recent articles that help elucidate the role ecosystem services play in the connection between ecological and human health risk assessment.

An update to EPA’s Generic Ecological Assessment Endpoints for Ecological Risk Assessment [118] includes generic ecosystem service (EPA/100/F15/005). An accompanying technical background paper described the science supporting the linkage between ecological structure and function, and ecological services to society (EPA/100/F15/004). The background paper is summarized by Munns et al. [119].

### 4.2. Conceptual Developments

Myers et al. [120] characterized the current research on health impacts of ecosystem services and described its limitations. Reis et al. [121] and De Laender and Janssen [122] discussed methods for integrating ecosystem services into existing risk assessment paradigms. These three studies are summarized in Table S10.

### 4.3. Methods and Applications

Articles in Table S11 explored the development of indices for quantifying ecosystem impacts on human health. Of note are the works of Ringold et al. [123] and Norman et al. [124], who demonstrated novel methods that enhance collaboration across multiple disciplines to evaluate health and well-being.

## 5. Promising Data Sources for Cumulative Risk Studies

The data and research generated from the National Children’s Study’s pilot effort, the Vanguard Study, could be a fruitful resource for secondary analyses of cumulative risk and children’s health; these data are archived and are available upon request [125]. Kim et al. [126] described the Mothers and Children’s Environmental Health (MOCEH) study in Korea, a prospective cohort of pregnant women and their children who will be followed to age 5. The most recent analysis to come out of the MOCEH study is from Bhang et al. [127] and examined relationships between maternal stress and infant developmental outcomes adjusting for prenatal heavy metal exposure. 

In addition to those publications that indexed available databases applicable for CRA [65,66,67], recent efforts have resulted in a public health exposome database and a toxic exposome database [64,128]. The exposome databases house an impressive breadth of information and could be useful for toxicologists, epidemiologists, biochemists, and omics-based disciplines interested in utilizing those resources for CRA applications. 

## 6. Discussion: Summary of the State of the Practice of CRA

### 6.1. Limitations

Our review captured ten years of literature focused on the risk analysis process but did not address cumulative risk management or decision making. This review summarizes this broad literature and is intended to introduce scientists and risk assessors to the variety of approaches that could be applied to understand cumulative exposures and risks. Readers seeking additional details in the tables in the Supplementary Materials may find that some papers appear connected, i.e., a research need described in one paper is addressed in another. This is not surprising since our search covered a ten-year period but we did not attempt to track or link papers in this way. Further, we did not undertake quality evaluation of the literature identified. This field of work is relatively new and includes many disciplines; there is no “standard” cumulative risk assessment and there are no “standard” methods. It may be possible to conduct evaluation in the future as the field develops; with experience it may be found that particular approaches and methods are shown to be valid and reliable for certain types of cumulative risk analyses. 

The studies included have addressed chemical mixtures and different combinations of stressors but many cumulative risk questions have yet to be tackled and our understanding of cumulative risks is limited. The search terms did not include particular stressors or some important issues such as exposures to pharmaceuticals through environmental media, or the impacts of genetically modified organisms on ecosystem services or our food system. Here, again, it is important to note that the studies included in this review are representative of conceptual or methodological advances and it is not the intent of the authors to evaluate the weight of evidence for the impacts of any particular stressor. It is also clear that, given the complexity of cumulative exposures, no matter what method(s) are applied uncertainty will remain. The core principles and practices of risk assessment including evaluation, quantification (when possible) and discussion of uncertainty and the values of prevention and harm reduction will continue to be essential for environmental health protection efforts addressing cumulative exposures and risks.

### 6.2. Summary and Highlights of Literature Reviewed

The pioneering work of Fox et al. [129] was an early application of cumulative risk methods to understand community health and investigate environmental justice concerns. The key features of this work were the assessment of a large mixture of Hazardous Air Pollutants (HAPs) and incorporating multiple health effects per HAP in a community-level study. Fox et al. looked at the correlations and associations between total, cardiovascular and respiratory mortality and HAP health risks at the census tract level in south and southwest Philadelphia. The investigators included some simple stratified analyses by white and nonwhite populations and used per capita income and percent nonwhite population as control variables in regression analyses. They used a GIS to produce descriptive maps of HAP risk scores. The main finding of the work was that increased HAP risk scores were associated with increased total and respiratory mortality [129].

The literature gathered for this review covered 2007 to 2016. Cumulative risk work in this time period includes significant advances in methods, research and thinking about complex environmental exposures and risks beyond that represented by Fox et al. [129]. Perhaps most important is the availability of measures of nonchemical stressors beyond census data (e.g., ways to represent the social context, instruments to assess chronic life stress, animal models of stress). GIS applications also are much more sophisticated, including the development of databases with chemical and nonchemical exposure information as well as health outcomes [47,48]. There now are examples in the literature of powerful statistical methods well suited for cumulative risk problems, including multilevel modeling that allows the incorporation of individual- and group- or place-level data. There are also several examples of Bayesian methods and applications of fuzzy set theory.

The role of CA and IA models in predicting combined effects from chemical mixtures is uncertain [106,110,130], although several articles agree that CA is best reserved for conservative effect estimation [6,107,109] or estimation at low concentrations [108]. Studies in both human health and ecological health tackled the alterations in chemical risks that could occur from global climate change [96,114,131]. McEwen and Tucker [132] recognized the need for advancing research on the biological pathways through which stress affects health and modifies the effects of toxicant exposure. Dulin-Keita et al. [79] and Zota et al. [71] provided insights into these pathways in their research. 

There was a noticeable trend over time towards a more holistic approach to assessing cumulative risks as recommended by Cutchin [13]. Hennig et al. [133] suggested diet become a part of the risk assessment paradigm as a critical modulator between environmental pollutants and health status. GIS applications from Salinas et al. [49] and Huang and London [50] used indices that included health status, economic, environmental and social datasets. Gennings et al. [62] extended the systems biology approach to human well-being using biomarkers and a Relative Wellness Index (RWI). AL as an intermediary element in health outcomes took on a prominent role in more recent studies of cumulative risk. A number of review articles pointed to a widening evidence base for cumulative risks, and investigation of links between social and environmental stressors [16,17,43,86,92]. 

Approaches linking ecological and human health risk assessments through ecosystems services also are promising. There is a large and growing evidence base for the influence of ecosystem services on human health [117] and existing research provides methods for quantifying this impact [123,134]. 

#### An Essential Reading List

Table S12 highlights the papers from this review that capture essential concepts, methods and new directions in research approaches and findings. By reading these papers, scientists new to the CRA field would find the conceptual bases and examples of studies that could fuel design and implementation of further and much-needed research. 

### 6.3. Types of CRA Studies Represented in Literature Reviewed (Stressor- or Effects-Based Assessments)

Menzie et al. [14] described two broad types of risk assessments, stressor- and effects-based assessments. Stressor-based assessments start with consideration of the stressors of concern, generally chemicals but increasingly nonchemical stressors as well. Effects-based assessments begin with identification of health effects of concern (e.g., high rates of cancer or childhood asthma). The majority of research identified in this review was stressor-based (A particular investigation may be initiated from concerns of certain health effects but eventually be presented as a stressor-based assessment in the peer-reviewed literature, perhaps to better conform to expectations of editors or peer-reviewers in environmental science fields). The standard risk assessment paradigm, with its focus on chemical stressors of regulatory concern, is likely a major contributor to the paucity of effects-based assessments. 

The Menzie et al. paper [14] and the NRC report on Cumulative Risks of Phthalates: The Tasks Ahead [7] may help researchers to see the value of, and way to execute effects-based assessments. Menzie et al. [14] described a systematic approach, including a step-by-step process for effects-based assessments. The main recommendation of the phthalates report, that common adverse outcomes should become a focus of CRA efforts, also is a call for effects-based assessments. 

### 6.4. Discussion of Literature Reviewed Following Recommendations from the National Research Council

The NRC’s Committee on the Health Risks of Phthalates recommended common adverse outcomes as the organizing principle for CRAs (not the more limited common mechanism approach). The committee recommended that cumulative assessments of common adverse outcomes take into consideration multiple types of stressors that act via multiple dissimilar mechanisms. 

Research on phthalates and other anti-androgens continues to be the primary example of risk assessment work addressing “common adverse outcomes” [10]. The literature included papers describing exposures to varied stressors and mechanisms (e.g., [135]). From the ecological risk assessment literature, Vidau et al. [116] and Langmead et al. [111] are relevant examples of studies of stressors that act via dissimilar mechanisms of action on outcomes of concern. 

The main recommendations of the Science and Decisions report [3] for CRA were the following:

- Consider methods and approaches from ecological risk assessment and social epidemiology.
- Increase the role of biomonitoring, epidemiology and surveillance data in CRA.
- Develop simpler analytic tools.
- Consider and apply data on vulnerability and susceptibility.

All of these points are addressed in the papers reviewed in the Conceptual Developments Section 2.2, Section 3.1 and Section 4.2, and to some extent in the research and examples reviewed, although much more can and will be done to develop these recommendations for CRAs. Many studies explored new approaches to using biomonitoring, epidemiology (and social epidemiology), and surveillance data (e.g., [61,62,71,74,136]). Among contributions from social epidemiology are indices of social context (e.g., neighborhood psychosocial hazards [68]) and studies that examine stressors such as parental or household stress on children’s health [81,82]. Many epidemiological studies were captured in this review, and the majority of these looked at respiratory outcomes associated with air pollution exposures acting in concert with nonchemical stressors (e.g., social context, area-level SES), as mentioned above (see, for example, [8,73,85,86,88]).

In terms of simpler analytic tools, EPA has invested in databases and GISs to facilitate CRAs, as reflected in papers by Barzyk et al. [66], Zartarian and Schultz [137], MacDonell et al. [67] and Zartarian et al. [138]. The GIS applications summarized above provide new ways to assess chemical, physical and social exposures and sometimes health outcomes. Many of these GIS applications also use surveillance data for CRAs. Susceptibility and vulnerability were considered by Alexeeff et al. [51], Hicken et al. [139], Young et al. [140], Norman et al. [124], in CRAs of chemical mixture exposure in children [39,40], and in a comprehensive review by Zeise et al. [22]. 

There was a substantial amount of cross-over between disciplines represented in the reviewed studies. For example, a social epidemiological study by Theall et al. [78] considered vulnerability using biomarker data. Papers presented in Section 4, Ecosystem Services, indicated work is under way to combine the efforts of ecological and human health risk assessment.

### 6.5. Discussion of Reviewed Literature in the Context of the Risk Assessment “Steps” 

Considering the literature reviewed as it relates to the “steps” of traditional risk assessment, problem formulation and exposure assessment were represented best. The numerous papers summarized in Conceptual Developments supported problem formulation by defining important concepts and frameworks for describing multiple contributors to health risk and suggested ways to operationalize them with data. 

The many GIS articles supported efforts at exposure assessment by combining data on chemical and nonchemical stressors so high-combination exposure sites can be identified. Alexeeff et al. [51], Salinas et al. [49], Huang and London [50], and Norman et al. [124] developed GIS methods for screening vulnerable communities, and these methods could be considered useful for hazard identification. Research results reporting dose-response relationships are represented (e.g., [71,72]), although some methods may need to be developed to allow incorporation of unconventional results (e.g., [62]) into risk assessments. Methods for synthesizing dose-response estimates reported for multiple stressors across multiple studies are described in Levy et al. [73], and greatly contributes to arguably one of the most difficult areas of CRA. For risk characterization, Landis et al. [112], for example, has demonstrated that Bayesian techniques can be successfully used improve characterization of uncertainty and provide more information relevant to decision-making. 

### 6.6. Progress Report: What Is the State of the Practice? 

#### 6.6.1. Weight of Evidence on Chemical Mixtures and Chemical and Nonchemical Stressors and Health Impacts

There is ample evidence from both human and ecological risk assessment fields that multiple-chemical and mixed-stressor exposures increase risks to health. On the ecological health side, for which the experimental evidence is strong, additive models routinely are applied and thought to provide conservative risk estimates [105,107]. The review of ecotoxicological studies by Holmstrup et al. [93] reported many examples of synergism and few examples of antagonism. Novel “omics” approaches in the ecotoxicological literature were used as a guide to clarifying the roles of CA and IA in effects at the molecular level, and provided further evidence of ecological health impacts.

#### 6.6.2. Exposures and Outcomes Examined So Far

Work on the effects of psychosocial stress of lead exposure and blood pressure [71,139], neighborhood disorder on AL in children [98,99], and reviews of the evidence of interactions between chemical and nonchemical stressors in both ecological and human health were captured in this literature review. 

The majority of work on, or related to, human health that has addressed both chemical and nonchemical stressors has focused on air pollution exposure and mortality as well as respiratory outcomes (see [8]). Some articles evaluated diet and nutrition as an important intermediate in exposure–disease relationships [5,74,133]. A few studies have examined lead exposure, nonchemical stressors and neurological outcomes (also reviewed in [8]). Notable work with chemical mixtures includes the following:

- Hendriksen et al. [58] looked at mixtures of methyl mercury, benzene and trichloroethylene on liver and kidney effects in rats.
- Navas-Acien et al. [141] looked at joint exposure to lead and cadmium and kidney function in a population study.
- Al Zabadi et al. [54] used urinary biomarkers to assess exposure and genotoxicity to some polyaromatic hydrocarbons and volatile organics in an occupational study.
- Zota et al. [57] looked at exposures to multiple polybrominated diphenyl ethers and measures of thyroid function in pregnant women.
- Pelallo-Martinez et al. [40] assessed genotoxic and hematological effects of exposure to chemical mixtures in children living in industrial areas using urinary biomarkers.

Phthalates and other anti-androgen mixtures research is directed at male reproductive development and neurological development [10,56]. Studies of pesticide mixtures included organophosphates and carbamates (acetylcholinesterase (AChE) inhibition) and anti-androgenic pesticides (see [25,26]). There were several chemical mixture CRAs conducted since 2014 (see Other Chemical Mixtures in Section 2.3.1), indicating that the evidence base for evaluation of risk in this area is robust and growing. 

The body of work on combined exposures and ecological health risks was not exhaustively reviewed here. A few papers documented studies of chemical mixtures from small two-chemical combinations to up to 15 chemicals, including pesticides and persistent organics. According to Holmstrup et al. [93], the majority of cumulative ecological risk studies of chemical and nonchemical stressors have looked at only two factors. Some studies demonstrated methods and models new to ecological health, for instance, a fish model of chronic stress or metabolomics approaches to mixture identification and investigation of effects [100,102,142]. A recent ecological risk study demonstrates integration of a combination of 12 chemical and nonchemical stressor using Bayesian modeling techniques [112]. Ecological CRA outcomes cover a full spectrum from individual and population outcomes (e.g., growth, survival, and population abundance) to the “omics” (genomics, transcriptomics, etc.). 

#### 6.6.3. Promising Complementary Approaches to CRA

In addition to the new directions for CRA identified by the recent NRC panels, two other promising approaches were identified in this review: health geography and HIA. Cutchin [13] suggests that a “new health geography” can complement approaches to social epidemiology that may tend toward a reductionist risk factor approach. The concepts of health geography contribute to a better understanding of places and offer ways to understand the dynamics of social, physical and chemical exposures [13].

Morello-Frosch et al. [143] suggest that HIA may be a good tool to address CRA questions. HIA features strong stakeholder involvement, and health is conceptualized across many determinants, including but not limited to environmental chemicals [144,145].

The adverse outcome pathway (AOP) conceptual framework maps connections between stressors, molecular initiating events, and adverse health outcomes [146]. The AOP framework is a multidisciplinary approach to evaluating risks and incorporates information from computational toxicology, in vitro studies, and animal studies. It provides a visual representation of toxicological mechanisms or modes of action linked to health effects of interest in a population. The AOP framework may be useful in CRAs to map pathways from stressors to outcomes and investigate levels of uncertainty associated with data that indicates linkages between important variables. Mapping AOPs can provide a way to evaluate multiple stressor-effect pathways, and it is envisioned that as AOPs and networks of AOPs are constructed and gathered into a knowledge database, human health and ecological risk assessment will benefit from its predictive capabilities [147].

Decision analysis is an additional resource that can help practitioners address qualitative, or less scientific aspects of CRA involving values and preferences, along with quantitative information [148,149].

### 6.7. Persistent Challenges in the Development and Practice of CRA

Identifying and fostering the most useful approaches in the area of nonchemical stressors appears to be a persistent challenge in the development of CRA for human health. Clougherty and Kubzansky [24] reviewed the complexities in measuring social stress, which include the following:

- Developing good measures and biomarkers of stress while being cognizant of the phases of the stress response.
- Considering the temporal relationships between pollution exposure and stress.
- Accounting for the spatial correlation among social and physical or chemical exposures.
- Factoring in pollution and pollution sources as psychosocial stressors.
- Understanding that area-level socioeconomic data reflect many complex exposures, and physiological stress may or may not be closely related to all of them.

Additional challenges facing practitioners in planning effective CRAs also include:

- Modifying CRA frameworks to work at the community, state, and federal levels [16,17].
- Identifying applicable data sources for the population of interest.
- Considering co-exposures that may be regulated by different agencies.
- Defining the scope of a CRA so that appropriate information from both ecological and human health risk studies can be effectively applied.

### 6.8. Research and Data Needs

Exposure assessment and dose-response are the quantitative inputs necessary to develop risk estimates. Work on identification of cumulative exposures is well-represented in this review. More work to improve our understanding of how to measure non-chemical stressor exposures and cumulative dose-response is needed. Many of the needs listed below address these research gaps. Findings and conclusions of several papers are reflected in the listing of research and data needs [5,8,14,24,93,96,104,120,150,151,152,153]. Research and data needs are organized into categories of human health, ecological health, and those that apply to both human and ecological health (including ecosystem services). Further research will help to identify the most useful of these approaches and indicators, and those that will be most effective in enhancing community involvement.

#### 6.8.1. Human Health

- Careful attention to the measures and proxy variables for social or psychosocial stress, and AL.
- Toxicological research using animal models of social stress, including attention to the human relevance of such models.
- Applications addressing exposures and health effects other than criteria air pollutants and related effects (mortality, respiratory diseases).
- Applications of GIS to examining health outcome questions/hypotheses (beyond using tools just to describe and identify areas of high combination exposure).
- Better understanding of short-term and long-term effects of exposure to nonchemical stressors.

#### 6.8.2. Ecological Health

- Prioritization of the most important mixtures or combinations of stressors.
- Testing of more complex mixtures beyond two-stressor experiments.
- Attention to timing and sequence of exposures.
- Focus on collecting chronic mixture toxicity data.
- Long-term experiments to incorporate climate change projections, including adaptive potential to climate stress; habitat shifts or reduction; microevolution; and comparative studies of climate tolerance in reference populations and toxicant-resistant populations.

#### 6.8.3. Human and Ecological Health

- Attention to susceptibility and vulnerability leading to the “cumulative dose–response.”
- Dose-response modeling with advanced methods, such as fuzzy set theory, Bayesian modeling, and multi-level modeling.
- Biomarkers and mechanistic studies; calls for further work in toxicodynamics.
- Focus on multiple human health impacts of ecosystem degradation.
- Investigation of health outcomes resulting from the interaction of multiple environmental changes.
- Exploration of human adaptation to environmental changes and how this mediates health outcomes.
- Better characterization of populations affected by ecosystem alteration.

## 7. Conclusions

This article focused on presenting and highlighting research and methods from scientific literature to advance method development for conducting CRAs, and offers a synthesis of studies from a number of disciplines to serve as a reference to those interested in advancing the field of CRA. To provide a state of the practice and an overview of current methods and tools for CRA, a literature search was conducted using terms selected through consultation with EPA staff, and found many studies of cumulative risk problems in the science and practice literature from 2007 to 2016. 

Studies contributing methods and applications for assessment of cumulative risks were gathered from varied subject areas including GIS, biomarkers, genetics, and “omics” research, social epidemiology, ecotoxicology, and climate change research. These studies supply evidence that health risks increase from combined exposures to chemical and nonchemical stressors or chemical mixtures. Important conceptual developments have been made in CRA and in disciplines supporting CRA (e.g., epidemiology and health geography) that strengthen the conceptual and theoretical frameworks necessary to improve characterization of cumulative risks. This article also identified many different disciplinary approaches and indices used to describe the social context for adverse outcomes. Staying abreast of new concepts and incorporating these concepts into CRAs will continue to be a challenge, however considerable improvements in the assessment of population health may be made as the evidence base for cumulative risks grows, measurements of social factors influencing risk are refined, and new concepts are incorporated into CRA’s framework.

## Figures and Tables

**Table 1:** Summary of highlighted papers—cumulative risk and human health.

Topics	Key Features Including Exposures, Outcomes, Methods
Conceptual Developments	Sociological theory as an approach for CRA.
	Global climate change expected to increase exposures to chemical contaminants as well as heat stress, water availability, nutritional changes.
	Review of allostatic load literature summarizing methods and findings of evidence associating AL with social, ethnic and economic disparities and health outcomes.
	Approaches to expand the use of epidemiological studies for CRA including: analyses of chemical mixtures; designs that inform cumulative dose-response.
	Organizational frameworks for CRAs.
	CRA in occupational health context.
	Definitions, data and indicators to support CRAs: vulnerability, susceptibility, health disparities, biological variability.
	Biomarkers, Health Impact Assessment, and Person-oriented modeling as methods for CRA.
	Review of literature on interactions of stressor combinations including physical and chemical stressors including air pollutants, pesticides and heat stress; radiation and chemicals; chemicals and infections, noise and chemicals.
Geographic Information Systems	Several GIS systems are described that combine environmental and socioeconomic data; social, physical and health data. Social data include census, crime, socioeconomic deprivation. Environmental data have included air pollution, wildfires, earthquakes, noise, road traffic, radiation/radon, water disinfection byproducts. Health data have included mortality, emergency department visits, hospital admissions, mental health measures, respiratory disease.
Biomarkers, Genetics, Omics	Genotoxicity and cancer risk of organic chemicals evaluated with urinary biomarkers.
	DNA damage from binary pesticide mixtures evaluated in peripheral blood lymphocytes.
	Case study of isoprostane biomarkers in raw sewage to evaluate community health status.
	Study using NHANES assessed association of body burden of 42 chemicals in blood or urine with a Relative Wellness Index reflecting organ system function.
	Study of phthalate mixtures and child development.
	Animal study of gene expression in liver and kidney examining interactions among exposures to methylmercury, benzene, and trichloroethylene.
	Prediction model for type 2 diabetes including genetic data, age, sex and BMI.
	Review of epigenetic findings for three different types of stresses, nutritional, psychosocial or toxics exposures (not in combination).
Measures and Models	Inventories of databases that can be used for CRA.
	Alternative approach to assessment of phthalate mixtures.
	Several frameworks/models for evaluating joint toxicity of chemical mixtures.
	Study that developed an animal model of chronic stress to look at health effects of stress and air pollutant exposure.
	Study that developed a model of combined psychosocial stressors.
	Inventory of testing methods for various chemical exposures.
	Social epidemiology study of neighborhood-level psychosocial stressors and lead exposure on cognitive function in adults.
	Study of chemical mixtures in air and food and associations with Disability-Adjusted Life Years.
	Two studies of AL as an effect modifier of lead exposure and blood pressure or as a factor in methylmercury exposure by race/ethnicity.
	Study using focus groups to assess place-based risk perceptions associated with industrial air pollution.
	Study developed an index combining ethnic and economic disparities with environmental hazards.
	Development of models of drug and chemical interactions focused on cytochrome P450 CYP3A4.
	PBPK/PD model framework to evaluate how poor nutrition affects internal dose of two organophosphate pesticides.
	Study demonstrated approaches for dose–response assessment for multiple stressor exposures.
Studies of Vulnerable Populations	Several studies of children: traffic related pollution and psychosocial stress; neighborhood stressors/disorder and serum cortisol; social position and lung and immune function, allergy; parental stress and children’s lung function or asthma.
	Residents near swine concentrated animal feeding operation, community-based participatory study of CAFO-related exposures, respiratory outcomes and stress.
	A national-level analysis found that disadvantaged communities experienced greater exposures and neurological, cancer and respiratory hazards from air pollutants.
	Several geographic/spatial models: screening tool using publicly available data; spatial model demonstrated at a community near an incinerator and Superfund site; “climate health justice” model including disease projections, treatment costs and social disparities for diseases with strong linkage to climate change.
	Study of psychosocial stress, blood lead and blood pressure in blacks versus whites.

**Table 2:** Summary of highlighted papers—cumulative risk and ecology or ecosystems.

Topics	Key Features Including Exposures, Outcomes, Methods
Conceptual Developments	Review of ecotoxicological literature on chemical, biological and physical stressors.
	Study of life-course and sequencing of exposure in crustacean.
	Description of NoMiracle project—developing methods for human and ecosystem health monitoring.
	Review of selected case studies examining climate change, toxicant exposure and ecosystem health.
Measures and Models	Study of biomarkers of exposure in fish to inform follow-up studies on health effects.
	Studies of binary mixtures of selected pesticides and antibiotics with metabolomics, proteomics, gene expression (earthworms, marine mussels, bacteria).
	Studies of complex mixtures (insecticides and/or herbicides or organic compounds) examining CA and IA dose-response models (crustacean, bacteria).
	Study of pesticide and heat stress on salmon.
	Study of mercury and other chemical and physical stressors in rivers (abiotic and biotic endpoints).
Studies of Ecological Areas or Populations	Application of Bayesian methods to model of different types of socioeconomic development on a large ecosystem.
	Experimental model of chronic stress in fish with exposure to fasting or heat.
	Study of zooplankton chemical and physical stressors.
	Review of ecological studies (aquatic and terrestrial plants and animals) that assessed dose-response models (CA, IA) of combinations of radiation and other stressors.
	Experiment on bees assessing infection and exposure to pesticides.
Conceptual Developments (Ecosystem Services)	Integration of ecosystem perspective into ecological risk assessment framework.
	Linking human health to ecosystem services.
	Adaptation of DPSEEA model; defining “ecological public health”.
Measures and Models (Ecosystem Services)	EPA Eco-Health Relationship Browser summarizes literature on positive effects of ecosystem services on human health.
	GIS tool to assess community access to ecosystem services.
	Framework to identify data indicating ecosystem impact on human well-being.
	Indices of human well-being and dependence on ecosystem services.

## Supplementary Materials

The following are available online at www.mdpi.com/1660-4601/14/4/389/s1, Table S1: Cumulative exposure and risk concepts over time, Table S2: Conceptual developments—human health, Table S3: Cumulative risk methods and applications for human health: GIS, Table S4: Biomarker, genetic and “Omics” studies, Table S5: Measures and models, Table S6: Cumulative risk studies of vulnerable populations, Table S7: Conceptual developments—ecological health, Table S8: Measures and models—ecotoxicology, Table S9: Examples of ecological cumulative risk studies, Table S10: Conceptual developments—ecosystem services, Table S11: Methods and applications—ecosystem services, Table S12: Papers exploring essential concepts, methods and new directions.

## Abbreviations

The following abbreviations are used in this manuscript:

AChE	acetylcholinesterase
AL	allostatic load
BMI	body mass index
CA	concentration addition
CAFO	concentrated animal feeding operation
CAOS	common adverse outcomes
CEQ	Council on Environmental Quality
CO	carbon monoxide
CRA	Cumulative Risk Assessment
DA	dose addition
DPSEEA	Driving Force-Pressure-State-Exposure-Effect-Action
DPSIR	Drivers-Pressures-States-Impact-Response
EFSA	European Food Safety Authority
EM	effect modification
EPA	U.S. Environmental Protection Agency
FEV1	forced expiratory volume in 1 second
GIS	geographic information system
H2S	hydrogen sulfide
HAP	Hazardous Air Pollutant
HIA	Health Impact Assessment
IA	independent action
LD50	lethal dose, 50%
mmHg	millimeter of mercury
MOA	mode of action
mRNA	messenger RNA
NAAQS	National Ambient Air Quality Standards
NEPA	National Environmental Policy Act
NHANES	National Health and Nutrition Examination Survey
NO2	nitrogen dioxide
NOx	nitrogen oxides
NRC	National Research Council
PAH	polyaromatic hydrocarbon
PBPK	physiologically based pharmacokinetic
PD	pharmacodynamic
PK	pharmacokinetic
PM	particulate matter
PM_10_	fine particulate matter (diameter ≤ 10 μm)
PM_2.5_	ultrafine particulate matter (diameter ≤ 2.5 μm)
RfD	reference dose
RWI	Relative Wellness Index
SEP	socioeconomic position
SES	socioeconomic status
TCDD	tetrachlorodibenzodioxin
TSP	total suspended particulates
VOC	volatile organic compound
