# Supplementary material for: An Overview of Literature Topics Related to Current Concepts, Methods, Tools, and Applications for Cumulative Risk Assessment (2007–2016)
Source: Int J Environ Res Public Health. 2017 Apr 7;14(4):389. doi: 10.3390/ijerph14040389 (PMC5409590; doi:10.3390/ijerph14040389)
Supplement: Supplementary file 1 [file ijerph-14-00389-s001.pdf]

Supplementary Materials: An Overview of Literature Topics Related to Current Concepts, Methods, Tools, and Applications for Cumulative Risk Assessment (2007–2016)

Mary A. Fox, L. Elizabeth Brewer and Lawrence Martin

Table S1. Cumulative exposure and risk concepts over time.

Reference or Source	Key Concept(s) or Definition(s) Introduced
National Environmental Policy Act (NEPA) of 1969 (P.L. No. 91-190)<br>Council on Environmental Quality regulations implementing NEPA [1]	"Cumulative effects" include exposure over time and additive, antagonistic and synergistic effects.
Guidelines for the Health Risk Assessment of Chemical Mixtures [2]	Chemical mixtures exposures can occur from multiple environmental sources or hazardous waste sites.<br>Formulas for the two primary models of joint action, dose or concentration addition (DA or CA) and response addition or independent action (IA).
Pesticides in the Diets of Infants and Children [3]	Aggregate exposure and risk considers exposure and risk associated with all pathways and routes of exposure to a single chemical.<br>Cumulative risk is the risk of a common toxic effect associated with concurrent exposure by all relevant pathways and routes of exposure to a group of chemicals that share a common mechanism of toxicity.
Executive Order (EO) 12989 "Federal Actions to Address Environmental Justice in Minority Populations and Low Income Populations"<br>EO 13045 "Protection of Children from Environmental Health Risks and Safety Risks"	Some populations may be more vulnerable and more highly exposed to environmental chemicals.
Phthalates and Cumulative Risk Assessment: The Tasks Ahead [4]	Recommended common adverse outcomes as a broader organizing principle for cumulative risk assessments than a common mechanism of toxicity.
Science and Decisions: Advancing Risk Assessment [5]	Found that EPA "cumulative risk" work to date had largely been limited to addressing chemical mixtures.<br>Recommended that EPA expand its cumulative risk efforts to include nonchemical stressors, population vulnerability and susceptibility.

Table S2. Conceptual developments—human health.

Reference	Main Points	Findings/Challenges/Next Steps/Possible Applications
**Akom [6]**	• Explores environmental exposures and educational outcomes.<br>• Written from a sociology/race theory/social epidemiology perspective.<br>• Describes the "eco-apartheid framework".	• Eco-apartheid framework also may be a useful conceptual framework for joint analysis of social and environmental inequities.
**Balbus et al. [7]**	• Discusses the potential for global climate change to alter the human health risks of chemical stressors.<br>• Describes potential effects of climate stress on exposure from major contaminate sources and vulnerable populations, as well as implications of climate change on current risk assessment and management practices.	• Net increase in exposure to contaminants likely.<br>• Climate change will affect fate and transport; types, amounts, and patterns of chemicals used; naturally occurring toxicant exposures (e.g., aflatoxin); and change in human vulnerability to stressors resulting from changes in diet, heat stress, and other factors.<br>• Future research requires a holistic approach and world-wide collaboration from many disciplines.
**Barzyk et al. [8]**	• Provides recommendations for consistent CRA approaches across community, state, and federal levels.<br>○ Methods are under development at each level, but there are no cross-cutting recommendations for best practices for all CRAs.<br>• Recommendations are organized by the three phases of CRA: planning, scoping, problem formulation; risk analysis; and risk characterization, interpretation, and management.	• Identifies relative risk ranking and other procedures as alternative strategies to estimate risk while advances are made in the science characterizing MOAs for multiple stressors.<br>• By comparing and contrasting CRA approaches at multiple levels, the authors identify components necessary to include in any CRA.
**Beckie [9]**	• Evaluates the empirical literature (1997–2012) on the relationships between allostatic load (AL), health disparities, and health outcomes.<br>• Describes allostasis, or the regulatory process of approximating biological functioning to environmental demands to preserve physiological stability, through a heuristic model of AL:<br>• Allostatic challenges/factors that shape response to stress (e.g., genetics, SES, behavioral factors, anxiety, childhood adversity) → perceived stress → primary mediators (e.g., cortisol, dopamine, interleukin-6) → secondary outcomes (e.g., blood pressure, waist-hip ratio) → tertiary outcomes (e.g., mortality, physical health).	• Although lack of homogeneity in AL measurements across studies made comparison difficult and resulted in inconsistent AL/health disparity/health outcome relationships, there was good evidence of relationships between AL, antecedents and tertiary outcomes.<br>• Further investigation is needed to identify the best methods for measuring AL.<br>• AL research is a promising resource for identifying interventions for reducing the impacts of biological dysregulation resulting from chronic stress.<br>• Early intervention may help reduce chronic illnesses and eliminate health disparities.
**Braun et al. [10]**	• Summarizes the challenges of studying the impacts of chemical mixture exposure from an epidemiological perspective.<br>• Proposes three questions future studies can address to help advance method development for better understanding of the effects of exposure to chemical mixtures on human health.	• Defined research questions are:<br>○ What are the health effects of individual chemicals within a mixture?<br>○ What are the interactions between chemicals within a mixture?<br>○ What is the health effect of cumulative chemical exposure?<br>• Questions can be applied to other mixtures including non-chemical stressors.

**Buzzelli [11]**	• Argues for using Bourdieu's [12,13] relational framework of social stratification—using social, cultural, and economic capital as a more nuanced approach to evaluating the psychosocial environment.<br>• Uses air pollution literature as an example of rigorous environmental science/epidemiology that so far has used only simple markers of socioeconomic position (SEP).	• The author cites a need for better data for all aspects of environmental justice studies (i.e., measures of physical and social environment and health outcomes).
**Checker [14]**	Presents:<br>• Argument for community participatory research.<br>• Role for anthropologists, e.g., on-the-ground assessments with refugees to properly characterize risk experienced from a number of different circumstances.<br>• Case study of Hyde Park area of Augusta, Georgia.	
**Clougherty and Kubzansky [15]**	Reviews:<br>• Biologic mechanisms through which acute and chronic psychosocial stress can affect health.<br>• Key issues for assessment of stress, air pollution exposure and respiratory health.<br>• Need for toxicological research with good animal models of social stress (e.g., rats, monkeys).<br>• Value of human experimental studies on acute and chronic psychosocial stress.	Issues with measuring psychosocial stress:<br>• Phases of stress process, biomarkers of stress response.<br>• Temporality of stress and pollution exposure.<br>• Spatial correlation among social and physical exposures.<br>• Exposure–response may be nonlinear and exhibit saturation effects.<br>• Pollution and pollution sources also are psychosocial stressors.<br>• SES is linked to many complex exposures; stress may reflect some but not all of these.
**Cutchin [16]**	• Describes how "new health geography" can contribute to understanding physical and social environments and inform social epidemiological studies.<br>• Illustrates several health geography concepts with a community case study in Texas City, Texas.	• Health geography offers explanations for how certain social and physical exposures come about.<br>• Health geography analyses will suggest different types of interventions than the "traditional" risk-factor type of epidemiological study.<br>• Concepts of health geography can strengthen social epidemiological approaches to questions of environment and health.
**deFur et al. [17]**	• Proposes a model for incorporating vulnerability in CRA.<br>• Includes individuals, communities, and populations as receptors.<br>• Includes physical and social environments in environmental conditions.<br>• Lists factors contributing to vulnerability across levels of environment and receptors.<br>• Draws from both human and ecological health risk concepts.	• Maintain focus of research effort on causes of vulnerability so that the causes can be addressed.
**Hennig et al. [18]**	• Argues for the incorporation of nutrition or dietary choices into the risk assessment paradigm as a critical modulator between environmental pollutants and health status.<br>• Describes studies demonstrating:<br>• Healthful nutrition or diet as an important buffer against chemical, physical, and biological stressors.<br>• Poor nutrition or diet increasing vulnerability to pollutants.	• Recommends diet and nutrition be considered in CRAs and be used as a tool for intervention and risk reduction.

**Howd [19]**	• Describes age and lifestage as susceptibility factors as well as determinants of differential chemical exposure.	
**Knol et al. [20]**	• Provides background on the use of conceptual frameworks as a tool to inform environmental health research or assessments.<br>• Describes three levels/types of frameworks and how each framework relates to issue framing (problem formulation), design (planning and scoping), execution (analyses), and appraisal (risk characterization and reporting).	
**Lentz et al. [21]**	• Describes potential for CRA approaches to be adapted to occupational scenarios<br>• Illustrates factors that may need to be considered to integrate risk from occupational and non-occupational settings using a case study of a hypothetical individual with hearing loss.	
**Levy [22]**	• Describes a framework for evaluating epidemiological studies as informative for CRA (particularly for dose–response).<br>• Identifies characteristics of an "ideal" epidemiological study for cumulative risk.	• Identifies the need to have:<br>○ Useful epidemiological studies for many cumulative risk questions.<br>○ Communication between epidemiologists and risk assessors to develop useful studies.<br>○ Approaches that can draw on both epidemiology and toxicology.<br>• Proposes that meta-regression will be useful to combine results of multiple studies.
**Linder and Sexton [23]**	• Argues for the need for theoretical frameworks to support "speculative" conceptual models that have been used in CRAs.<br>• Discusses three theoretical frameworks that each support corresponding families of conceptual models:<br>○ Social determinant framework from the World Health Organization.<br>○ Health disparity framework from the Centers for Population Health and Health Disparities.<br>○ Multiple stressor framework from Morello-Frosch and Shenassa.	• Using established theoretical frameworks will lead to improvements in the characterization of cumulative risks.

**Menzie et al. [24]**	• Describes "Phased Approach" for CRA:<br>o Conceptual model<br>o Screening step<br>o Assess individual stressors<br>o Assess combinations of stressors<br>• Includes:<br>o Tables to illustrate methods for effects- and stressor-based assessments.<br>o Listing of multivariate statistical tools for addressing multiple stressors.<br>o Geographic Information System (GIS) applications.<br>• Matrix-based approaches (Relative Risk Model).	• A useful "how-to" guide for study design and implementation.
**Morello-Frosch et al. [25]**	• Briefly reviews evidence of several factors of interest in CRA:<br>o Health disparities by race/ethnicity.<br>o Inequalities in environmental hazards and exposures by race/ethnicity and low SES.<br>o Biological susceptibility by age, genetics, and preexisting health conditions.<br>o Social vulnerability—how social constructs of race and class can amplify environmental exposures. For example, low SES, poor physical environment, and other factors contribute to psychosocial stress (see Clougherty and Kubzansky [15]<br>o Limitations of current risk assessment approaches.	• Suggests that Health Impact Assessment (HIA) may be a better tool for characterizing cumulative impact than risk assessment because it is better able to deal with a lack of scientific information by using a diverse array of qualitative and quantitative evidence for analysis.
**Segal et al. [26]**	• Using lead and psychosocial stress as an example, describes a framework for evaluating chemical-nonchemical interactions and options for incorporating interactions into risk assessments.	• Evidence is supportive of interactive effects of lead and psychosocial stress on neurodevelopment.<br>• Additional research to understand effects of nonchemical stressors at the biological level is needed.
**Sexton [27]**	• Examines effects-based CRA and discusses vulnerability-based CRA as an additional useful alternative to examining health risks in communities or other defined populations.<br>• Reviews existing tiered and phased approaches for CRA.	• Characterizes the difficulties of development methods for and implementing CRAs.<br>o Need for risk assessments addressing more realistic exposure scenarios.<br>o Complicated by a lack of mechanistic understanding of complex exposure–response relationships.<br>• Suggests focus on developing tiered/phased approaches and appropriate science policies to address uncertainties and variation in levels of available information for analyses.

**Sexton and Linder [28]**	• Argues that CRA is a logical and impartial method for informing decision-making about environmental justice issues. CRA can:<br>○ Identify critical environmental mixtures of chemical and nonchemical stressors,<br>○ Determine the nature and magnitude of relevant cumulative exposures for the population of interest,<br>○ Describe key interaction mechanisms and related health outcomes for high-priority mixtures.<br>• Discusses a set of typical critiques that are representative of views challenging risk assessment's application to environmental justice communities, and asserts these critiques foreshadow advancement in CRA method development.	• Recommends the following approaches to developing and advancing CRA:<br>○ Identify high-priority communities, populations or places;<br>○ Undertake a coordinated research effort;<br>○ Use conceptual model and research data to assess cumulative risks; and<br>○ Modify, revise, or reject the conceptual model on the basis of research findings.
**Smith et al. [29]**	• Describes exposomics as the study of all nongenetic factors that contribute to disease, measured by the influence of small molecules in the body on biological pathways that lead to adverse health effects.<br>• Discusses the application of exposomics to CRAs in vulnerable populations using existing frameworks.<br>○ Describes the public health exposome concept [30].<br>○ Defines "stressogens" as chemicals that disrupt stress response pathways; evaluation of these chemicals can contribute to a more holistic approach to assessing health risks.	• Suggests high priority populations for study: recent migrants, highly exposed low socioeconomic groups, and pregnant women.<br>• Begin by applying exposomics in populations previously studied using cumulative risk methods.
**Rider et al. [31]**	• Provides and discusses frameworks that can be used to include nonchemical stressors into CRAs.<br>• "Person-oriented modeling" is a computer simulation that models exposure patterns and uncertainty that is then repeated to create population descriptions.<br>• A combination of EPA's conceptual models for metals and the weight of evidence framework from the Agency for Toxic Substances Disease Registry.<br>• Presents case study of benefits and risks of fish consumption using a comparative dietary risk framework.	• Take steps to facilitate quantification of information for inclusion in CRA:<br>• Standardize terminology.<br>• Identify sources of information.<br>• Compile good quality causal models that relate joint exposures to chemical and non-chemical stressors to human health effects.<br>• Achieve a better understanding of long-term effects of exposure to non-chemical stressors.<br>• Shift focus from reference doses (RfDs) and No Observed Effect Levels to ranges of values.<br>• Use semiquantitative methods (e.g., Hazard Index informed by weight of evidence) for immediate inclusion of nonchemical stressors in risk characterization.

**Rider et al. [32]**	• Reviews evidence of interactions between physical and chemical stressors:<br>• Sunlight and air pollutants<br>• Air pollutants, pesticides, and thermal stress<br>• X-rays and chemicals<br>• Chemicals and infectious disease<br>• Noise and chemicals	• Include physical stressors in CRA because they modify the toxicity of chemical stressors. Link physical factors (e.g., temperature, noise at site) to environmental sampling for further assessment of physical and chemical interactions.<br>• Use a systems-based approach to predict interactions<br>• Combine simulation approaches with monitoring data for incorporation of physical stressors.<br>• Add estimates of the magnitude of modification of interactions to the CRA process.<br>• Develop more case studies.
**Ryan et al. [33]**	• Reviews biomarkers.<br>• Discusses attributes of ideal biomarkers.<br>• Offers framework to illustrate how multiple stressors relate to biomarkers of exposure, susceptibility, and effect.<br>• Describes two ways to use biomarkers:<br>• Disaggregating contributions of multiple stressors to a disease process. Identify contributing sources and stressors using biomarkers of exposure, susceptibility, and effect.	Challenges:<br>• Will require large amounts of related data and well-developed biologic mechanisms to fully understand any particular biomarker.<br>• No fully developed example exists.
**Sexton and Linder [34]**	• Reviews existing chemical mixture methods.<br>• Presents Gee and Payne-Sturges and Shenassa and Morello-Frosch multistressor conceptual models.<br>• Identifies several indices for assessing health and SES disparities (see [35]).	Next steps to advance cumulative risk methods and practices:<br>• Identify high-priority communities/populations/places.<br>• Undertake a coordinated research effort.<br>• Use conceptual model and research data to assess cumulative risk.<br>• Modify/revise/reject conceptual model on the basis of research findings.
**Zeise et al. [36]**	• Reviews approaches for describing interindividual variability and susceptibility within a "source-to-outcome continuum".<br>• Adjustment factors.<br>• Pharmacokinetic (PK), pharmacodynamic (PD), and physiologically based pharmacokinetic (PBPK) modeling approaches.<br>• Discusses new data on biological variability.<br>• Reviews in vitro, in vivo, and in silico models.<br>• Discusses the use of Genome-Wide Association Studies in clinical and epidemiological studies to investigate susceptibility though genetic analysis.	• Integrate emerging data streams into in silico models for decision-making applications.

Table S3. Cumulative risk methods and applications for human health: GIS.

Reference	Main Points	Findings/Challenges/Next Steps/Possible Applications
**Aagaard-Hansen et al. [37]**	• Provides a public health rationale for GIS-based surveillance systems that combine information on population health, physical, chemical and biological exposures and social determinants of health (as demonstrated by Basara and Yuan [38], below).	• Valuable as a descriptive tool to identify high-risk populations.
**Andrey and Jones [39]**	• Describes a GIS developed to evaluate selected environmental and socioeconomic hazards in Vancouver, Canada.<br>• Based on Canadian Census for 1986, 1996 and 2001.<br>• Environmental hazards: earthquake-induced liquefaction, wildfires and noise pollution.<br>• Many demographic and socioeconomic variables.<br>• Applies Principle Components Analysis—results in groups of variables with similar spatial patterns as components, which in turn suggest the spatial structure of the data.	• Social and environmental hazard exposures were dynamic over time.<br>• Authors found inconsistent patterns of minority- or income-based environmental inequity over the 15-year period studied.<br>• Descriptive study—no analysis of health outcomes; no chemical exposures.<br>• Application of Principle Components Analysis may be of interest as a method.
**Basara and Yuan [38]**	• Describes and illustrates a GIS-based data management system incorporating social, physical and health outcome data sets.<br>• Could be used to identify and group communities with similar characteristics.	• These types of databases then can be used in studies to generate and test cumulative exposure and outcome hypotheses.<br>• Limited to existing data sources (e.g., EPA's Toxic Release Inventory, census, hospitalization data).

**Briggs et al. [40]**	• Assesses associations between multiple measures of socioeconomic deprivation and five sets of environmental pollutants in England:<br>○ Index of Multiple Deprivation includes: income, employment, education, health, living environment, barriers to housing and services, and crime.<br>○ Components of the health indicator: Years of potential life lost, comparative illness and disability ratio, emergency hospital emissions, and adults with mood or anxiety disorders.<br>○ Stressors include: road traffic, industry, electromagnetic frequency (EMF) radiation, disinfection byproducts in drinking water, and radon.<br>○ "Exposure" measured in terms of proximity to source, emissions and concentrations.	• This study assessed many bivariate associations but not combined impacts of SES and environment on health.<br>• It confirmed the existence of environmental inequities associated with socioeconomic deprivation.<br>• Stronger associations occurred with crime, living environment and health (contingent components of deprivation), rather than with causative components such as income, employment or education.<br>• For environmental concentrations, strongest associations were found between SES and air pollutants rather than proximity to source or emissions.<br>• Overall, the associations between environmental factors and SES were "weak, subtle and complex"; the strength of association varied depending on metrics used.<br>• Areas of "triple jeopardy" (socioeconomic deprivation, poor health and poor environment) were limited.<br>• Problems of confounding by SES in area-level environmental epidemiological studies were likely limited because of the weak associations between environmental conditions and SES in this analysis.
**Huang and London [41]**	• Develops a Cumulative Environmental Vulnerability Assessment comprised of three indices to identify environmental justice communities in the San Joaquin Valley, California, through spatial analysis:<br>○ Cumulative Environmental Hazard Index<br>○ Social Vulnerability Index<br>○ Health Index	• Indices include health status and social vulnerability indicators beyond income and race.<br>• Can be used to focus resources on the most vulnerable communities.<br>• Limited by the accuracy and availability of the data sets, and the geographic unit of analysis (census block group).
**Maclachlan et al. [42]**	• Describes an Internet-based GIS developed to investigate relationships between health, air quality and socioeconomic factors in Hamilton, Canada.<br>• Reports on a pilot test and focus group evaluation for public health professional users of the system.	• No analytical results of environment-health-SES relationships.<br>• An interesting tool that could be replicated in other geographic areas.

**Salinas et al. [43]**	• Demonstrates the Human Security Index as a useful tool for understanding and correlating socioeconomic and environmental stressors with race/ethnicity at the county level.<br>• Defines cumulative risk burden by indicators from Economic, Environmental, and Social "Fabrics" (e.g., poverty, incarceration rate, high ozone days).<br>• Uses GIS to identify statistical associations between risk burden, ethnicity, and place.	• Index useful for identifying the relative contributions of impacts from chemical and nonchemical stressors on community health.
**Shmool et al. [44]**	• Demonstrates a GIS approach for examining spatial patterns among 29 administrative indicators of social stress, and their combined effects with air pollution on health in New York City, using New York City Community Air Survey (NYCCAS) data.<br>• Also performs an ecologic analysis investigating effect modification between nitrogen dioxide (NO_2) and childhood asthma Emergency Department (ED) visit rates by social stressors.	• Social stressors were not consistently correlated, nor were they correlated with other indicators of SEP.<br>• Social stressors appeared in geographically distinct patterns, which were characterized by the following factors:<br>○ Violent crime and physical disorder<br>○ Crowding and poor access to resources<br>○ Noise disruption and property crimes<br>• In the ecologic analysis, these factors were differentially associated with area-average NO_2 and childhood asthma ED visits.
**Tornero-Velez et al. [45]**	• Investigates environmentally relevant chemical co-occurrence using a biogeographical method.<br>• Assumes chemical occurrence at specific locations is not random; characteristics of a location favor certain chemical combinations.	• Biogeography methods can be used to help risk assessors identify mixtures of concern and prioritize efforts.
**Wang and Chen [46]**	• Uses a GIS-based modeling approach for assessment of air pollution.<br>• Considers multiple pollutants (chemical mixtures).<br>• Uses fuzzy aggregation modeling to quantify uncertainty.	
**Zartarian et al. [47]**	• Designed the Community-Focused Exposure and Risk Screening Tool, a Web-based GIS to support community-level risk research and environmental justice efforts.	• Beta-version currently being pilot-tested.

Table S4. Biomarker, genetic and "Omics" studies.

Reference	Main Points	Findings/Challenges/Next Steps/Possible Applications
**Al Zabadi et al. [48]**	• Assesses overall genotoxicity of worker exposures to polyaromatic hydrocarbons (PAHs) and VOCs with integrated and nonspecific urinary biomarkers.	• Urinary biomarkers reflected higher exposure for sewage workers than for office workers and corresponded to increased lifetime cancer risk.
**Das et al. [49]**	• Assesses three, two-pesticide mixtures on viability and DNA damage in cultured human peripheral blood lymphocytes:<br>o Monocrotophos and carbofuran<br>o Endosulfan and monocrotophos<br>o Endosulfan and carbofuran<br>• Evaluates viability with a standard cytotoxicity dye exclusion assay.<br>• DNA damage included chromosomal aberrations and comet assay.	• Authors found that combinations of pesticides showed synergism with regard to reduced cell viability and DNA damage in human peripheral blood lymphocytes in vitro.<br>• Authors suggest that these types of assays may serve as biomarkers of exposure or susceptibility.
**Daughton [50]**	• Proposes use of raw sewage to gauge community health (Sewage Chemical Information Mining).<br>• Detection of biomarkers of exposure and disease in sewage can provide a measure of the aggregate health status of individuals in a local population.<br>• Uses isoprostanes (biomarkers of systemic oxidative stress) as an example.	• Potential:<br>o Shows aggregate health status, health trends, and possible health disparities for communities.<br>o Allows health comparisons among distinct populations.<br>o Aids in causal inference for exposure disease relationships.<br>o "Health checks" for entire communities.
**Gennings et al. [51]**	• Investigated the link between body burden of 42 chemicals and well-being using National Health and Nutrition Examination Survey (NHANES) biomonitoring data and a holistic index.<br>o 42 chemicals were divided into 6 mechanistic groups that may affect health through different MOAs.<br>• Novel method for assessing multiple chemicals acting through multiple pathways that may cause multiple adverse effects (a systems biology approach).<br>• Developed Relative Wellness Index (RWI) to measure the function of multiple organ systems.<br>o RWI is predictive of mortality.<br>o RWI indicates the overall wellness of the subject by detecting conditions that are outside of the normal range for healthy organ system function.	• Negative associations were found between the body burden index and RWI in 3 of 6 mechanistic groups, and 5 of 6 groups when the body burden index was weighted.<br>o Suggests that exposure to environmentally relevant concentrations of chemicals in five of the groups will have adverse effect on well-being.<br>• Found evidence of interactions between mechanistic groups (antagonistic, greater than additive).<br>• Limitation: Biomonitoring data, collected at a single point in time, do not allow a determination of temporal relationships between exposure and disease.
**Whyatt et al. [52]**	• Studies maternal urinary phthalate metabolites and child development.<br>• Assesses biomarkers of exposure and clinical effects.<br>• Suggests that prenatal phthalate exposures decrease mental and motor development.	• New work on the health effects of phthalate exposures.

**Bonefeld-Jorgensen et al. [53]**	• Reviews recent biomonitoring literature on the combined effect of serum POP mixtures on the activity of hormone receptors as a risk factor for human health.	• Conclude that POPs biomagnify up the food chain, bioaccumulate in humans and animals, are potential endocrine disruptors and carcinogens, and can cause adverse human health effects.<br>• Suggest epidemiological molecular-genetic studies to further observe effects in human beings and animals.
**Hendriksen et al. [54]**	• Interactions of methylmercury, benzene and trichloroethylene on mRNA (messenger RNA) expression in rat liver and kidney were studied by microarray analysis.<br>• The three chemicals were selected on the basis of their different MOAs.<br>• Animals were dosed with low and high doses of the individual chemicals, all two-chemical combinations and the three-chemical mixture for 14 days.	• In the two-chemical combinations, the compounds had strong antagonistic effects on each other's gene expression changes, however, the mixtures affected the expression of "novel" genes that had little or no effect from the individual compounds.<br>• Authors conclude that the results suggest a shift from compound-specific responses to a more generic stress response to mixtures.<br>• Most effects observed in microarray analysis were not detected in classical toxicological evaluation of tissues.<br>• This type of study moves toward development of biomarkers of joint toxicity.<br>• The findings ultimately may lead to the design of interaction models for most known toxicants.
**Miyake et al. [55]**	• Developed a prediction model for type 2 diabetes.<br>• Included 11 genes as well as age, sex and body mass index (BMI).	• Perhaps this model could serve as a tool to explore chemical exposure in addition to genetic and physiological factors.
**North and Martin [56]**	• An overview of gene-environment studies<br>• Examples highlight obesity research with environmental factors defined as diet, physical activity, stress and other diseases.	• A framework for consideration of chemical exposures as well.
**Thayer and Kuzawa [57]**	• Reviews epigenetic findings related to nutritional stress, psychosocial stress, and toxicant exposure.<br>• Considers each type of exposure separately.	• A resource for identifying types and measures of epigenetic changes to incorporate into cumulative risk studies.

Table S5. Measures and models.

Reference	Main Points	Findings/Challenges/Next Steps/Possible Applications
**Barzyk et al. [58]**	• Identifies more than 70 EPA databases, data management, data display and GIS applications that can provide information for community-based CRAs.<br>• Databases and computational tools were developed by various EPA programs and reflect the particular program focus and data.<br>• Useful for issue identification, hazard identification.	• Typically more than one database is needed to fully evaluate all environmental media or sources of concern in a community.<br>• Most produce dose estimates but not risk characterization metrics.<br>• Difficulty accessing and using these technologies.<br>• Lack of nonchemical stressor data.<br>• Lack of risk-ranking capabilities.
**Borgert et al. [59]**	• Proposes Human Relevant Potency Threshold (HRPT) approach as a more scientifically tenable alternative to the phthalates report (NRC [4]) recommendation.<br>• Tested the concept recommended by the phthalates report that CRA should apply DA for all chemicals with common adverse outcomes (CAOS).<br>• Found the DA-CAOS model to be flawed after comparing its predictive capabilities to human clinical and epidemiological data.<br>• The proposed HRPT approach integrates parts of the DA-CAOS concept at doses near the lower limit of the observable effect range. Below this threshold, IA is recommended.	• Applying the approach to phthalates, authors found HRPT was more suitable to assess risk than DA by itself.
**Boyd et al. [60]**	• Demonstrated a novel in vitro method for predicting effects of chemical mixtures using a PD response.<br>○ Test of a concept that cellular interpretation of exposure is sufficient to predict interaction effects instead of relying on the chemical's MOA.<br>• Exposed human hepatocellular carcinoma-derived cells to deguelin, potassium cyanide, and staurosporine.	• Found the cellular response approach effective in identifying mixture interactions based solely on dose (rather than MOA).
**Chen [61]**	• Proposes an isobologram model for quantal dose–response data.	• Extension of isobologram methods for assessing joint action of chemical mixtures.
**Clougherty et al. [62]**	• Used an animal model of chronic social stress for toxicological study of effects of air pollutants.<br>• Social stress induced by putting young male rat in home cage of dominant male rat for 20 minutes.<br>• Biomarkers of physiological changes were indicative of potential disease mechanisms (susceptibilities).<br>• Environmental exposure was to concentrated urban air (dose–response).	• Stressed animals displayed higher average C-reactive protein, tumor necrosis factor-α, and white blood cell counts.<br>• Findings consistent with epidemiological findings that chronic stress may alter respiratory responses to air pollution.<br>• This model may help identify (and potentially quantify) biological mechanisms of differential susceptibility.<br>• This model may help quantify the combined stress and chemical response for dose–response assessment.

**Evans et al. [63]**	• Study on cumulative risk of exposure to low to moderate levels of psychosocial (e.g., workload, social support) and physical (e.g., noise, crowding) stressors in an occupational setting.<br>o Cumulative risk concept adapted from developmental psychology.<br>o Data self-reported.<br>o No chemical exposures evaluated.	• Identified linear trends between stressor exposures and outcomes (e.g., fatigue, psychological distress, physical health).<br>• Additive model is a drawback considering physical and psychosocial risk factors can interact.<br>• May nonetheless prove a useful example for evaluating the cumulative impacts of exposure to low levels of nonchemical stressors.
**Glass et al. [64]**	• Example of social epidemiological study of neighborhood-level stressors, lead and cognitive function in adults.<br>• Developed and used an indicator of neighborhood psychosocial hazard (from city and U.S. Census data sets) that includes the following:<br>o Social disorganization<br>o Public safety<br>o Physical disorder<br>o Economic deprivation<br>• Used multilevel statistical models.	• Neighborhood psychosocial hazards exacerbated adverse associations of tibia lead in three of seven cognitive domains (language, processing speed, and executive functioning).
**Glei et al. [65]**	• Examined the theory of AL in nationally representative longitudinal study in Taiwan<br>• Study questions:<br>o Do chronic stressors predict physiological dysregulation?<br>o Is that relationship moderated by characteristics of the individual and his/her social environment?<br>o Does perceived stress mediate the relationship between stressors and dysregulation?<br>• Developed a physiological dysregulation score from 16 biomarkers in blood or urine reflecting neuro-endocrine, immune, cardiovascular and metabolic function.	• Findings were consistent with theory of AL.<br>• Relationship between life challenges and physiological dysregulation was weak.<br>• Evidence of lower resilience (stress buffering), e.g., low social position, weak social networks, and poor coping were associated with greater physiological consequences.
**Hertzberg et al. [66]**	• Proposed four steps to evaluate chemical mixtures for consistency with DA:<br>o Determine how well the combined prediction model (i.e., the dose additive prediction formula) matches data from the single chemical models.<br>o Evaluate the fit of the mixture model to the mixture data.<br>o Evaluate agreement between the combined prediction model and the mixture data.<br>o Evaluate consistency between the combined prediction model and the mixture model.	• Four-step approach to evaluating mixtures is reasonable for a screening level assessment, but not for predicting mixture effects.<br>• More research using physiologically based toxicokinetic models would be helpful.

**MacDonell et al. [67]**	• An updated (as of early 2013) inventory of Internet-accessible tools for CRA.<br>• Describes how the tools can be used for CRA.<br>• Organized in tables by main phases of the risk assessment process:<br>○ Planning, scoping, problem formulation<br>○ Environmental fate and transport<br>○ Exposure analysis<br>○ Toxicity analysis<br>○ Risk and uncertainty characterization	
**Marshall et al. [68]**	• Proposed a method for examining whether a chemical mixture found in the environment is sufficiently similar to a mixture used in toxicity testing.<br>• Developed a similar mixture risk indicator to link available exposure data to sufficiently similar mixtures toxicology data.<br>• Used pesticide mixture exposure data at child care centers and dose–response data from an animal study of a similar mixture as an illustrative case study.	• Using the reference value from mixture toxicity data as a proxy for the RfDs for the observed pesticide mixtures at child care centers is a valid method for evaluating risk.
**Medina-Vera et al. [69]**	• Survey of environmental detection or measurement methods such as test kits for numerous analytes in water or indoor air, and for mold and lead on surfaces.	• Not all kits provide quantitative results.<br>• Quantitative methods are more costly.
**Navas-Acien et al. [70]**	• Examined effects of lead and cadmium exposure on kidney function measures in NHANES.	• This epidemiological study showed that those exposed to both lead and cadmium were more likely to have reduced kidney function.<br>• Study used national survey database, and biomarkers of exposure and effects. Measurements were taken at single point in time, inferring cause-effect relationship between exposure and outcome is limited.
**Ragas et al. [71]**	• Chemical mixture assessment considering particulate matter (PM), several VOCs and six food pesticides.<br>• Case example of hypothetical urban environment.<br>• Used Disability Adjusted Life Years as health outcome measure for selected chemicals.	• Recommendations:<br>○ Need to develop person-oriented exposure models to reflect normal daily activities.<br>○ Need better mechanistic understanding of cumulative effects beyond experimental investigation of antagonism/synergism.<br>○ Need methods to screen and prioritize stressors for inclusion in a CRA.

**Scholze et al. [72]**	• Developed a method for applying DA to mixtures of partial agonists using a toxic unit extrapolation approach.<br>• Validated with a mixture of estrogenic chemicals using an assay measuring the proliferation of human epithelial breast cancers.<br>o Estimation of cumulative internal exposure.	• Toxic unit extrapolation approach accurately predicted responses.<br>• Method can be applied to other pollutants.
**Silva et al. [73]**	• Evaluated CA as a model to predict effects of estrogenic mixtures including endogenous and synthetic steroidal hormones, pesticides, cosmetic additives and phytoestrogens (for a total of five mixture experiments).<br>• Mixtures were tested with the E-Screen (breast cancer cell proliferation assay).<br>• Considered findings in a context of regulatory applications.	• Effects of two of the mixtures were accurately predicted by CA.<br>• In three other cases, CA slightly overestimated findings.<br>• Authors found that increased metabolism of steroidal estrogens likely contributed to the antagonistic deviations from CA.<br>• Deviations from CA were small, leading the authors to conclude that CA remains a reasonable model to predict combination effects of estrogenic chemicals with the endpoint of cell proliferation.
**Zota et al. [74]**	• Using NHANES data, assessed how AL affects the relationship between lead exposure and blood pressure in middle-aged adults.<br>• First study to use AL to assess the effects of chronic stress on chemical exposure (others have used proxy measures like SES or self-reported stress levels).	• High AL is associated with an increase in adverse effects of lead on blood pressure.<br>• Limitations of this study include those inherent in cross-sectional study design, residual confounders, and issues selecting biomarkers representative of AL.<br>• Results suggest need for additional research on how psychosocial stress modifies the effects of chemical stressors in vulnerable populations.
**Kondo et al. [75]**	• Using data collection from focus groups in Philadelphia, PA, explores the role of place-based elements on risk perception associated with industrial air pollutants.	• Identifies three factors that must be addressed for effective community-based assessments:<br>o How community identifies with area and industry<br>o Highly perceptible stressors such as odors and abandoned sites<br>o Fear of displacement and lack of social control<br>• Assessments must also be sensitive to community fear and uncertainty about the research process itself to be successful.
**Bobb et al. [76]**	• Used Bayesian kernel machine regression (BKMR) to study mixtures.<br>o Health outcome is regressed on a flexible function of the chemical mixture components that is specified using a kernel function.<br>o Uses epidemiological and toxicological studies to illustrate.	• Has advantages over traditional frequentist approaches<br>o BKMR more fully captures uncertainty in the exposure–response function.<br>o Improved evaluation of mixture components.

**Su et al. [35]**	• Development and application of Cumulative Environmental Hazard Inequality Index (descriptive index of SES inequalities and cumulative environmental hazard).<br>• Index has two components:<br>o Measure of ethnic and socioeconomic inequalities.<br>o Estimates of cumulative environmental hazards.<br>• Example presented includes data from census (% nonwhite population and % population below 2× federal poverty line) and selected air pollutants (including cancer risk estimates for diesel PM).	• A descriptive index combining multiple stressors.<br>• Useful to identify high-risk areas.<br>• Could also include positive attributes (e.g., available green space, access to supermarkets).
**Tan et al. [77]**	• Describes potential of PK and PD models to understand effects of chemical mixtures.<br>• Some examples included solvents and pesticides.	• Emphasis has been on PK aspects.<br>• Need more PD work.
**Tie et al. [78]**	• Evaluated and suggested parallel use of a combination of several models to investigate interactions of drugs and chemicals that are inhibitors of cytochrome P450 CYP3A4 isozyme.	• Three models were suggested: computational molecular docking, structure–activity relationship, and spectral data-activity relationship.
**Wason et al. [79]**	• Developed a framework for a PBPK/PD model to evaluate the impact of poor nutrition on the internal dose of chlorpyrifos and acetylcholinesterase (AChE) inhibition in urban low-income children.<br>• Method and “proof of concept” application to introduce nonchemical stressors into the CRA paradigm.	• The combination of stressors is likely to increase risk of AChE inhibition in children from an urban low-income neighborhood.<br>• The PBPK/PD model allowed quantitative characterization of impacts of a chemical and nonchemical stressor on at PD outcome.<br>• The framework could be extended to include multiple stressors with same MOA.
**Levy et al. [80]**	• Demonstrated three meta-analytic approaches to dose–response development for multi-stressor assessments.<br>o A literature-based meta-analysis of the differential toxicity of fine particulate matter components.<br>o A structural equation modeling approach to an effects-based CRA of hypertension risk factors.<br>o A discrete event simulation model simulating the effect of changes in the built environment on environmental exposures and asthma outcomes.	• Emphasized the need for and importance of advanced analytical methods to synthesize evidence from multiple disciplines for CRAs.
**Evans et al. [81]**	• Characterized exposure to lead, methyl mercury and AL (i.e., chronic stress) using data from the 2003–2004 NHANES.<br>• Examined race/ethnicity as a predictor of joint exposure to lead and methyl mercury.	• Chronic stress modified the association between elevated joint lead/methyl mercury exposure and race/ethnicity.

Table S6. Cumulative risk studies of vulnerable populations.

Reference	Main Points	Findings/Challenges/Next Steps/Possible Applications
**Alexeeff et al. [82]**	• Developed a screening methodology for assessing cumulative impacts in 30 diverse California communities.<br>• Used zip code boundaries to delineate communities and publicly available data for indicators of exposure, sensitive populations, SES factors, public health and environmental effects.	• Useful tool for facilitating consideration of cumulative impacts in environmental justice communities.<br>• Also a significant contribution to the GIS literature.
**Bevc et al. [83]**	• Develops a spatial model including socio-demographics, perceived and objective exposure measures, and home-grown food consumption.<br>• Reviews proximity-based environmental justice research methods.<br>• Example community was near an incinerator and landfill (Superfund site):<br>o Environmental exposures were derived from an ash deposition model, years of residence, home-grown produce, well-water and local fish consumption.<br>o Psychological well-being was assessed with the Impact of Events Scale (relates to stress avoidance and intrusive stress) and the Depression Scale.<br>o Used hierarchical modeling.	• Found that socio-demographics, perceived and objective exposure measures, and home-grown food consumption were significant predictors of physical health and psychological well-being.<br>• A simple proximity-based exposure metric was not significantly associated with health.
**Chen et al. [84]**	• Presents results of a study of traffic-related air pollution and stress interactions as predictors of asthma outcomes in children.<br>• Sampled NO_2 at 116 sites, then used land-use regression to predict concentrations for participants.<br>• Psychosocial stress over the previous 6 months assessed with the University of California Los Angeles Life Stress Interview.<br>• Biomarkers: cytokines, IL-5, IgE, eosinophil counts.<br>• Clinical measures: parent and child interviews on symptoms; child diaries; Peak Expiratory Flow Rate taken twice daily for 2 weeks.	• Higher chronic stress was associated with higher inflammatory profiles as pollution levels decreased.<br>• NO_2 and stress interactions were found for child diary symptoms, parent-reported symptoms and Peak Expiratory Flow Rate declines over time.<br>• Conclusions:<br>o Stress-pollution interactions predicted biologic and clinical outcomes.<br>o Higher chronic stress made children vulnerable to asthma exacerbations when pollution exposure was more modest.

**Dulin-Keita et al. [85]**	• Conducted a longitudinal study of ethnically diverse children to evaluate effects of neighborhood stressors on serum cortisol levels.<br>• Focused on the direct effects of neighborhood socio-environmental conditions on physiological outcomes.<br>• Included body fat composition in analysis; known to affect cortisol levels and has not been considered in previous studies.	• Neighborhood disorder was predictive of higher serum cortisol in European American children, and was predictive of lower serum cortisol levels in African American children.<br>• Alteration of cortisol levels in either direction may lead to adverse health outcomes.
**Hicken et al. [86]**	• Evaluated effect of psychosocial stress, as measured by depressive symptoms, on the association between blood lead levels and blood pressure in blacks versus whites using NHANES data.<br>• A study of differential vulnerability to chemical exposure between blacks and whites.	• Black-white disparity shown in positive association between blood lead levels and blood pressure in groups with high depressive symptoms.<br>○ Blacks had 5.6 mmHg (millimeters of mercury) increase in blood pressure with each doubling of blood lead levels.<br>○ Whites' blood pressure increased 1.2 mmHg.<br>• No disparity was seen in low depressive symptoms groups.
**Hoffmann et al. [87]**	• Cross-sectional study of childhood social position, environmental exposures and lung function, allergy and immune function.<br>• Social position indicators: nationality, immigration background, parental education, household income, unemployment in family.<br>• Environmental exposures:<br>○ Total suspended particulate<br>○ Environmental tobacco smoke<br>○ Unfavorable living conditions (near roadway and/or damp residence)	• Socially disadvantaged children experienced higher exposures and were more likely to have unfavorable living conditions.<br>• Health outcome findings were complex and some were counter-intuitive; socially disadvantaged children were less likely to report allergic and respiratory diseases but more likely to have abnormal lung function in clinical testing.<br>• Explanations for the counterintuitive findings included selection and reporting bias and biologic interactions.<br>• Limitations: used secondary data analysis, did not evaluate neighborhood contextual effects, and did not have adequate sample size for stratified analyses.
**Islam et al. [88]**	• Describes a study of parental stress by pollutant interactions on children's lung function in a prospective cohort.<br>• Perceived parental stress served as a proxy for children's psychosocial stress.<br>• Traffic-related pollutants: nitric oxide, NO_2 and nitrogen oxides (NO_x) were estimated (from measurements) for homes and schools.<br>• Lung function was assessed by trained staff.	• Children from high-stress households had greater lung function deficits related to NO_x at home and school than those from lower stress households.<br>• No significant NO_x effects observed in children from low-stress households.<br>• Conclusion: A high-stress home environment was associated with increased susceptibility to lung function effects of air pollution.

**Pearlman [89]**	• A brief review of asthma epidemiology studies that are addressing community and neighborhood social factors.<br>• Refers to the work of Wright et al. [90].<br>• Social factors may increase risk or increase resilience.	• Multilevel models allow the assessment of clustering at the individual and neighborhood levels.<br>• Study noted issues with measurement of neighborhood social context, including:<br>○ Do you look only at the primary residence or other places as well?<br>○ How do you account for changes over time?
**Schulz et al. [91]**	• Conducted a study to examine the effects of neighborhood poverty on AL controlling for household poverty.<br>• Tested to see if self-reported psychosocial stress mediates the association.	• Found neighborhood poverty to be positively correlated with AL.<br>• Relationship mediated by perceived social stress, but not by health behaviors (e.g., smoking, diet).<br>• Connected SES to adverse health outcomes using physiological indicators.
**Shankardass et al. [92]**	• Describes a prospective cohort study of parental stress as effect modifier of traffic-related air pollution exposure and child asthma incidence.<br>• Air pollution exposure was modeled.<br>• Data on stress and other exposures were collected by survey.<br>• Doctor-diagnosed new asthma outcome was reported by parents at 3-year follow-up.	• Children of parents with high perceived stress were at higher risk of new-onset asthma associated with pollution.<br>• Similar findings from stress and maternal smoking in pregnancy (but small numbers of participants in this category).<br>• Authors suggest that high-stress home environment increases susceptibility to pollution and *in utero* tobacco smoke for asthma incidence.
**Theall et al. [93]**	• Investigated the connection between salivary telomere length and exposure to neighborhood level stressors in children.<br>• Used telomere length as a biomarker of AL.<br>• First study to seek a cellular response to community-level psychosocial stress; also could be categorized as a novel biomarker study.	• Children living in neighborhoods characterized as having high levels of disorder had shorter telomere lengths.<br>• Findings support hypothesis that neighborhood level stressors cause adverse biological responses in early life.<br>• Potential confounders are bacterial contamination and DNA degradation related to saliva sampling.<br>• Interventions should be focused as early in life as possible to prevent health disparities.
**Erickson and Arbour [94]**	• Explored the mechanistic pathways between exposure to particulate air pollution and adverse pregnancy outcomes.<br>• Also investigates similarities to mechanisms where SES contributes to adverse pregnancy outcomes.<br>• Review literature showing interactive effects between air pollution and stressors associated with SES.	• Evidence suggests that various social and environmental exposures can cause common adverse pregnancy outcomes.<br>• Suggests using multilevel models in future epidemiological studies to explore the biological effects of the social and physical environment.

**Wing et al. [95] (description) Horton et al. [96] Schinasi et al. [97] (results)**	• Describes an epidemiologic study of respiratory outcomes and stress in communities proximal to swine concentrated animal feeding operations (CAFO).<br>• Academic-community partnership.<br>• 2-week longitudinal study.<br>• Air monitoring.<br>• Participants used diaries and personal respiratory monitors for outcome data collection.<br>• Assessed coping style using "John Henryism" Active Coping Scale.	• Increased acute eye irritation with each unit increase in odor, H_2S and PM_{10}.<br>• Odor and H_2S during the previous 12 hours were associated with irritation and respiratory symptoms.<br>• Increase in 12-hour $PM_{2.5}$ was associated with wheezing and declines in forced expiratory volume in 1 second (FEV1).<br>• Endotoxin was associated with increased sore throat, chest tightness and nausea.<br>• Odor, H_2S and PM_{10} were associated with stress and negative mood.<br>• Individuals who perceived that they had more control over their environment found malodor more stressful than those who perceived they had less control.<br>• Authors suggest odor be included in studies of environmental injustice.
**Wing et al. [98]**	• Conducted a community based participatory repeated measures study on adults living near a swine CAFO.<br>• Each participant measured their blood pressure and reported levels of hog odor after sitting outdoors at scheduled times for two weeks.<br>• Authors measured ambient levels of H_2S and PM_{10} during same time period.	• Increases in malodorous air pollution from CAFO were associated with increased blood pressure.<br>• SES, BMI, medical history, etc. were not confounders because participants served as their own controls.<br>• Chronic effects of exposure not evaluated.<br>• Swine CAFOs are commonly located near low-income minority communities; acute exposures to hog odors contributes to health disparities.
**Young et al. [99]**	• Demonstrated the value of social epidemiology in evaluating differential exposure to hazardous air pollutants (HAPs) related to neighborhood-level socioeconomic deprivation.<br>• Assessed neurological, cancer, and respiratory air pollutant hazards associated with the Townsend Index of Socioeconomic Deprivation.	• Found positive associations between air pollutant hazards and the Townsend Index at the census-tract level.<br>• Found clear evidence of differential vulnerability in disadvantaged communities.
**Schule and Bolte [100]**	• Systematically reviews multilevel modeling studies that consider both neighborhood SEP and factors of the objective built environment simultaneously in to evaluate their effects on individual health.	
**McDonald et al. [101]**	• Provides a scalable "climate health justice" model for assessing and projecting incidence, treatment costs, and sociospatial disparities for diseases with well-documented climate change linkages.	• Demonstrates a low-cost and easily implementable tool that can be utilized for geographic analyses of disparate health impacts associated with climate change.<br>• Model can help inform development of public health intervention strategies.

Table S7. Conceptual developments—ecological health.

Reference	Main Points	Findings/Challenges/Next Steps/Possible Applications
**Holmstrup et al. [102]**	• Reviews ecotoxicological literature on studies of chemical and natural stressors such as temperature, desiccation, oxygen depletion, pathogens.<br>• Most studies include only two factors (e.g., one chemical and heat).<br>• Many examples of synergistic interactions, a few antagonistic interactions.	• Research needs to develop this area:<br>o Comparative studies to identify the most potent combinations of natural and chemical stressors.<br>o Examine the sequencing of exposures.<br>• Limitation: These are largely short-term studies.
**Jansen et al. [103]**	• Explores the concepts of life-course and sequencing of exposures in a case study of *Daphnia*.<br>• *Daphnia* populations were exposed to carbaryl and developed resistance to the pesticide.<br>• These populations were then exposed to biological or predation stresses and fitness/survival was assessed.<br>• Possible mechanisms investigated with genome/transcriptome methods.	• Carbaryl-resistant *Daphnia* were more susceptible to Pasteuria ramosa (bacterial endoparasite).<br>• Carbaryl-resistant *Daphnia* were not more vulnerable to predation stress.<br>• Main point: Past exposures to pesticides are important and should be considered when evaluating current stressors.
**Lokke [104]**	• Describes the NoMiracle Project.<br>• Focuses on human and ecological risk and chemical mixtures.<br>• Develops methods for modeling fate and transport/exposure.<br>• Develops methods for assessing effects of chemical mixtures, including some in vitro testing of human cell lines.<br>• Uses assessment and management models, for example:<br>o Ecological vulnerability assessment<br>o Human respiratory health risk<br>o Uncertainty and variability tool	• Project directed at development of new methods for assessing risks to human and ecological health from chemical mixtures.<br>o Methods and model development included:<br>▪ Databases<br>▪ "Omics" systems<br>▪ In vitro screening systems<br>• Author states need for better understanding of mechanisms.<br>• Author states importance of timing and sequence of exposures.<br>• Concludes that research should focus on the receptor rather than on particular stressors or combinations of stressors.

**Moe et al. [105]**	• Discusses the joint effects of global climate change and toxicant exposure on the health of ecosystems.<br>• Outlines the challenge in ecotoxicology of predicting the effects climate stress will have on population responses to toxicants.	• Recommends research on adaptive potential to climate stress using genetic variability and correlation analysis; microevolution; comparative studies of climate tolerance in reference populations and toxicant-resistant populations.<br>• Recommends long-term experiments to incorporate climate change projections.<br>• Notes a need to integrate climate change into ecotoxicological modeling focusing on non-additive interactions between climate stressors and chemicals, population vulnerability, resilience, and adaption to same.

Table S8. Measures and models—ecotoxicology.

Reference	Main Points	Findings/Challenges/Next Steps/Possible Applications
**Al-Salhi et al. [106]**	• Identified the xenometabolome (xenobiotics and metabolites) that accumulate in fish exposed to wastewater treatment works effluent.<br>• Roaches were then exposed to the identified mixtures to investigate metabolite responses.	• Metabolite biomarkers can be used to monitor exposure to complex chemical mixtures in fish and inform follow-up studies on health effects.
**Baylay et al. [107]**	• Assessed effects of binary mixtures on *Lumbricus rubellus* using CA and IA models along with a metabolomics based approach.<br>• Exposed to imidacloprid and thiacloprid (similar MOAs), as well as chlorpyrifos and nickel (dissimilar MOAs).	• Imidacloprid/thiacloprid mixture:<br>o Exhibited additivity but metabolite changes indicated distinct effects.<br>o Independent joint effect also exhibited at higher exposure concentrations.<br>• Chlorpyrifos/nickel mixture<br>o Effects confirmed dissimilar MOAs.<br>o Found to be more toxic than predicted.<br>• Metabolomic analysis revealed more complexity in mechanisms of action than assumed in CA and IA.
**Dondero et al. [108]**	• Investigated the toxicodynamics of a neonicotinoid insecticide mixture on marine mussels.<br>• Tested imidacloprid and thiacloprid.<br>• Methods included gene expression and proteome profiles.	• Each insecticide had distinct toxicodynamics as shown by different transcriptomic and proteomic profiles, and also had opposite trend of AChE.<br>• Findings generally met principle of IA at the biomarker level despite having the same mode-of-action.<br>• High-throughput molecular data may help shed light on mechanistic pathways of individual chemicals when mixture studies yield counterintuitive results.

**Garcia-Reyero et al. [109]**	• Used *Daphnia magna* as biomonitoring tool for complex mixtures.<br>• Exposure to mixture of munitions contaminants.<br>• Response was measured in the change in gene expression using polymerase chain reaction assays and high-density microarrays.	• Prediction of gene expression change after chemical mixture exposure is possible to a limited extent.<br>• Limited to nonadditive effects, contaminants in similar chemical class.
**Wang et al. [110]**	• Integrated fuzzy CA-IA model demonstrated in mixture experiments with 12 organic chemicals representing four different MOAs.<br>• Model uses molecular structural information and fuzzy set theory to characterize the degree of similarity or dissimilarity of MOAs in a mixture.<br>• This model was successfully applied to mixtures with similar or dissimilar MOAs.<br>• This study tests the model in mixtures that combine similar and dissimilar MOAs.	• Study showed that this model had very low predictive error in comparison to the two-stage prediction model.<br>• Mechanism of action/MOA information is not needed for this approach.
**Zhang et al. [111]**	• Demonstrated the Uniform Design and fixed-ratio ray design experimental techniques.<br>• 15 pesticides (insecticides and herbicides) in 18 mixtures.<br>• Evaluated the observed versus predicted concentration-response functions by both CA and IA.	• Uniform Design effectively simulated numerous complex mixtures.<br>• CA accurately predicted the combined toxicity of pesticide mixtures.<br>• CA and IA predictions were very similar.
**Zou et al. [112]**	• Developed a method for testing the chronic toxicity of mixtures using antibiotics and *Vibrio fischeri*.<br>• Model is combination molecular docking-based and quantitative structure–activity relationship receptor library.	• Limited to binary mixtures, chemicals with similar structures.<br>• Future ecological research should focus on collecting chronic mixture toxicity data (as opposed to acute).
**Landis et al. [113]**	• Conducted a risk assessment using the Bayesian Network Relative Risk Model (BN-RRM) to calculate the ecological risks from exposure to mercury and other chemical and physical stressors in the South River and upper Shenandoah River area.	• Three main findings are reported:<br>○ Risk varied by location, type and quality of habitat, and exposure to stressors.<br>○ Risk to abiotic endpoints was greater than risk to biotic endpoints. Including both endpoints allowed comparisons among endpoints that represented various stakeholder values.<br>○ Mercury was the regulated stressor for the South River, but was not the only stressor influencing risk. Other ecological and habitat stressors contributed to risk and should be considered as part of risk management plans.

**Dietrich et al. [114]**	• Examined the effect of acute and sublethal exposures to the organophosphate pesticide malathion and elevated temperature on Chinook salmon.<br>• Study demonstrates impacts of anthropogenic stressors in the ecological context.	• There was an observed increase in mortality in salmon exposed to both malathion and elevated temperature.<br>• Salmon exposed to malathion under normal temperatures were not at risk of increased mortality.<br>• Demonstrated an interaction between co-occurring stressors, and that risk may be underestimated if stressors are evaluated individually.

Table S9. Examples of ecological cumulative risk studies.

Reference	Main Points	Findings/Challenges/Next Steps/Possible Applications
**Langmead et al. [115]**	• Assessed models of the effects of socioeconomic development on the marine environment of the Black Sea.<br>• Informed by DPSIR, a framework used to organize social and ecological information and map pathways from human activities to changes in ecological states, with available data sets used as indicators of drivers, stressors and states.<br>• Applied Bayesian belief networks.<br>• Developed four models for the socioeconomic development of post-Soviet countries surrounding the Black Sea along lines of consumerism vs. community and autonomy vs. interdependence.<br>o National Enterprise<br>o Local Responsibility<br>o World Markets<br>o Global Community<br>• Indicators of the marine environment included fish catches, hypoxia and sea grass habitat, among many others.	• Two of the four development models (the "National Enterprise" and "World Market" scenarios) increase pressures on the Black Sea environment.<br>• Currently, no policy tools address the important ecological health drivers identified in this study.<br>• Bayesian belief networks were useful in combining different types of information.<br>• A novel approach; ecosystem modeling does not typically consider larger socioeconomic processes.
**McConnachie et al. [116]**	• Experimentally elevated cortisol levels in fish to mimic chronic stress and then exposed the fish to either heat or fasting stress.	• Cortisol elevation increased short-term and long-term physiological vulnerability to subsequent stressors.
**Stampfli et al. [117]**	• Investigated the combined effects of an insecticide (esfenvalerate, a chemical stressor) and hydrological fluctuation (a physical stressor) on zooplankton communities.<br>• Extreme hydrological disturbance is predicted from global climate change, which could modify the effects of chemical exposures in aquatic communities.	• Hydrological stress increased the sensitivity of the zooplankton to the insecticide.<br>• Effects were additive, detection of a synergistic relationship was not possible in the experiment.

**Vanhoudt et al. [118]**	• Reviewed 35 ecological studies of the combined effects of radiation and other stressors (e.g., chemical, temperature, salinity) in aquatic and terrestrial plants, terrestrial animals (rats and mice), and aquatic animals.	• In all but one study, CA and IA were not predictive of combined effects.<br>• Interactions were instead categorized as “positive interactions” (i.e., potentiation, synergism) or “negative interactions” (i.e., antagonism) or no interaction.
**Vidau et al. [119]**	• Describes an experiment with bees infected with *Nosema ceranae* and also exposed to sublethal doses (1/100th of LD_{50} (lethal dose, 50%)) fipronil or thiacloprid.<br>• Explores the hypothesis that *N. ceranae* infection could modify the bees’ detoxification capacity.	• Observed increased mortality for infected bees also treated with insecticide as compared to infected and uninfected controls.<br>• Increased mortality was not “strongly linked” to decreases in detoxification systems.<br>• An example of impacts of biological and chemical stressors.
**Vieira and Guilhermino [120]**	• Studied the effects of PAHs and temperature stress on the marine algae *Tetraselmis chuii*.<br>• Experimental conditions set to mimic increase in temperatures caused by global climate change and increase in marine organism exposures to petrochemicals caused by ocean transport of oil (potential spills) and other products.	• A 5 °C increase in temperature caused increased toxicity of PAHs to the algae.

Table S10. Conceptual developments—ecosystem services.

Reference	Main Points	Findings/Challenges/Next Steps/Possible Applications
**De Laender and Janssen [121]**	• Propose integration of the ecosystem perspective into ecological risk assessment (European Union perspective).	• Identifies 5 key areas of research that are necessary for extending ecological risk to the ecosystem level: resource competition, predation, chemical effects on biodiversity, chemical effects on ecosystem services, and chemical mixture effect quantification at the ecosystem level.
**Myers et al. [122]**	• Discusses current research on ecosystem services and links to human health and identifies areas of improvement.<br>• Current literature is "patchy" and important relationships are not completely characterized.<br>• Aggregate ecosystem alterations are affecting multiple dimensions of human health and requires a systematic approach to characterizing health outcomes.	Limitations of current research:<br>• Focuses on only one human health impact of ecosystem degradation (e.g., infectious disease) instead of multiple health outcomes.<br>• Inadequate investigation of health outcomes resulting from the interaction of multiple environmental changes (e.g., climate change in conjunction with resource scarcity).<br>• Inadequate exploration of human adaptation to environmental changes and how this may mediate health outcomes.<br>• Inadequate characterization of populations affected by ecosystem alteration (i.e., differential vulnerabilities in poorer communities).
**Reis et al. [123]**	• Proposes a conceptual model to integrate human health and environmental impact analysis.<br>• The ecosystems-enriched Drivers, Pressures, State, Exposure, Effects, Actions model merges ecological health and human well-being through ecosystem services.<br>• Describes the "ecological public health" concept, which calls for integration of the social, economic, environmental, and public health realms.	• Model shows potential for stakeholder engagement and needs refinement, but is a good starting point for holistic assessment of ecological and human health.

Table S11. Methods and applications—ecosystem services.

Reference	Main Points	Findings/Challenges/Next Steps/Possible Applications
**Jackson et al. [124]**	• Developed a Web-based application called the Eco-Health Relationship Browser (http://www.epa.gov/research/healthscience/browser/introduction.html).<br>• Data from 169 articles included in the browser and reflects the weight of evidence on the positive and buffering that effects ecosystem services have on human health.	• Limitation: Does not include studies of the associations between the built environment and health and well-being.
**Norman et al. [125]**	• Developed a GIS for ecosystem services using the Soil and Water Assessment Tool and mapped socioeconomically disadvantaged populations near the U.S.-Mexico border using a Modified Socio-Environmental Index.<br>• Spatially correlate community access and exposure to ecosystem services.<br>• Method for identifying environmental justice communities using ecosystem services instead of chemical exposures.	• Found that border communities carry disproportionate environmental burdens resulting from ecosystem service degradation.
**Ringold et al. [126]**	• Developed a framework for identifying data that can serve as an indication of ecosystem impact on human well-being.<br>• Argued for the use of "final" ecosystem services, or services that are directly consumed or enjoyed by humans, as the most robust link to human well-being.<br>• Defined final ecosystem services as "biophysical features, quantities, and qualities that require little further translation to make clear their relevance to human well-being".<br>• Applied a six-step process to streams to demonstrate the framework.	• Framework aids in moving toward quantification of ecosystem services on well-being.
**Smith et al. [127]**	• Proposed a modeling structure for evaluating the impacts of ecosystem services on human well-being.<br>• Categorized indicators from existing well-being indices into domains (e.g., education, health, social cohesion) for use in a well-being index for the United States.	• Recognized the difficulties in using well-being indices in policy, but proposed model aggregates and incorporated well-known and accepted measures and methodologies.
**Yang et al. [128]**	Proposed an index of dependence on ecosystem services:<br>• Quantifies patterns of human dependence on ecosystem services.<br>• Higher value equals higher dependence on ecosystem services and increased vulnerability to ecosystem degradation.	• Found that the poor and those with less control of natural capital are more dependent on ecosystem services.<br>• The method can be used to identify interventions for poverty alleviation.

Table S12. Papers exploring essential concepts, methods, and new directions.

Reference	Category	Contribution to Cumulative Risk
**Alexeeff et al. [82]**	Method: GIS for Environmental Justice	Screening method for communities disproportionately exposed to social, economic, and environmental stressors.
**Bevc et al. [83]**	GIS Application and Methods	Example of an approach to assessing multiple factors causing mental and physical health problems.
**Briggs et al. [40]**	GIS Application	Analyzes correlations of SES and environment with health status (most GIS work was descriptive).
**Clougherty and Kubzansky [15]**	Concepts	Explains the concepts and challenges of measuring social stress.
**deFur et al. [17]**	Concepts	Highlights the concepts of susceptibility and vulnerability and issues a call to remain focused on identifying causes of susceptibility and vulnerability rather than mitigating factors.
**Gennings et al. [51]**	Method: Biomarkers	Presents a method for linking body burden of chemicals to human well-being.
**Glass et al. [64]**	Methods: Epidemiology	Exemplifies social epidemiological approach and a measure of social context.
**Glei et al. [65]**	Methods: Epidemiology	Assesses biological impacts of stress; findings support theory of AL.
**Hendriksen et al. [54]**	Methods: "Omics" for chemical mixtures	Illustrates the limitations of classic toxicology and advantages of "omics" technologies.
**Hicken et al. [86]**	Method: Differential Vulnerability	Assessment of differential effects of psychosocial stress on the association between chemical exposure and health outcome.
**Islam et al. [88]**	Methods: Epidemiology	Example of impacts of household stress on child pulmonary function in response to exposures to NO_x.
**Knol et al. [20]**	Concepts	Reviews the development and use of conceptual models underpinning research in environmental public health.
**Landis et al. [113]**	Method	Assesses ecological risk from multiple stressors using Bayesian methods.
**Levy et al. [80]**	Methods: Dose–response	Demonstrates three different meta-analytic techniques for dose-response development.
**Linder and Sexton [23]**	Concepts	Presents theoretical frameworks that can be used to support CRA conceptual model development.
**McConnachie et al. [116]**	Method: AL	Ecological example of interactions between chronic stress and environmental stressors.
**Menzie et al. [24]**	Concepts and Process	Presents systematic processes for conducting cumulative risk research and offers reviews of statistical models and analytical approaches appropriate for different types of risk questions/study circumstances.
**North and Martin [56]; Thayer and Kuzawa [57]**	Methods: Gene-Environment and Epigenetics	Presents the building blocks for further research and application of gene-environment and epigenetic studies to cumulative risk investigations.
**Rider et al. [31]**	Concepts	Discusses frameworks and models for integrating nonchemical stressors into CRA.
**Rider et al. [32]**	Review of Concepts	Reviews evidence for interactions between chemical and nonchemical stressors.
**Ringold et al. [126]**	Method: Ecosystems and Human Health	Provides a framework for identifying indicators of ecosystem service impact on human well-being.
**Sexton [27]**	Concept	Reviews and synthesizes existing CRA concepts and frameworks, and presents a path forward for implementation.
**Theall et al. [93]**	Method: Children's Health	Provides an example of a biomarker study of cellular response to neighborhood-level stressors.
**Tornero-Velez et al. [45]**	GIS Method	Biogeographical method for identifying environmentally relevant chemical co-occurrences.
**Wang and Chen [46]; Wang et al. [110]**	Methods	Applications of fuzzy set theory to ecological dose–response and air quality index for selected criteria air pollutants.
**Wason et al. [79]**	Method: PBPK	Demonstrates a method for introducing nonchemical stressors into the risk assessment paradigm.
**Wing et al. [95]; Horton et al. [96]; Schinasi et al. [97]**	Methods: Epidemiology	Example of community-based participatory research, 2-week longitudinal environmental epidemiology study, application of individual level stress, and mood instruments.
**Zota et al. [74]**	Method: AL	Provides a method for assessing how AL affects the relationship between a chemical stressor and a health outcome.

References

1. Council on Environmental Quality (CEQ). Considering Cumulative Effects under the National Environmental Policy Act. Available online: http://energy.gov/sites/prod/files/nepapub/nepa_documents/RedDont/G-CEQ-ConsidCumulEffects.pdf (accessed on 29 November 2016).
2. U.S. Environmental Protection Agency. Guidelines for the Health Risk Assessment of Chemical Mixtures. Available online: http://cfpub.epa.gov/ncea/cfm/recordisplay.cfm?deid=22567 (accessed on 29 November 2016).
3. National Research Council (NRC). *Pesticides in the Diets of Infants and Children*; National Academies Press: Washington, DC, USA, 1993.
4. National Research Council (NRC). *Phthalates and Cumulative Risk Assessment: The Tasks Ahead;* National Academies Press: Washington, DC, USA, 2008.
5. National Research Council (NRC). *Science and Decisions: Advancing Risk Assessment*; National Academies Press: Washington, DC, USA, 2009.
6. Akom, A. Eco-apartheid: Linking environmental health to educational outcomes. *Teach. Coll. Rec.* **2011**, *113*, 831–859.
7. Balbus, J.M.; Boxall, A.B.A.; Fenske, R.A.; McKone, T.E.; Zeise, L. Implications of global climate change for the assessment and management of human health risks of chemicals in the natural environment. *Environ. Toxicol. Chem.* **2012**, *32*, 62–78.
8. Barzyk, T.; Wilson, S.; Wilson, A. Community, state, and federal approaches to cumulative risk assessment: Challenges and opportunities for integration. *Int. J. Environ. Res. Public Health* **2015**, *12*, 4546–4571.
9. Beckie, T.M. A systematic review of allostatic load, health, and health disparities. *Biol. Res. Nurs.* **2012**, *14*, 311–346.
10. Braun, J.M.; Gennings, C.; Hauser, R.; Webster, T.F. What can epidemiological studies tell us about the impact of chemical mixtures on human health? *EHP* **2016**, *124*, doi:10.1289/ehp.1510569.
11. Buzzelli, M. Bourdieu does environmental justice? Probing the linkages between population health and air pollution epidemiology. *Health Place* **2007**, *13*, 3–13.
12. Bourdieu, P. The social space and the genesis of groups. *Theory Soc.* **1985**, *14*, 723–744.
13. Bourdieu, P. Distinction: A social critique of the judgement of taste. *J. Econ. Sociol.* **2005**, *6*, 25–48.
14. Checker, M. "But I know it's true": Environmental risk assessment, justice, and anthropology. *Hum. Organ.* **2007**, *66*, 112–124.
15. Clougherty, J.E.; Kubzansky, L.D. A framework for examining social stress and susceptibility to air pollution in respiratory health. *Environ. Health Perspect.* **2009**, *117*, 1351–1358.
16. Cutchin, M. The need for the "new health geography" in epidemiologic studies of environment and health. *Health Place* **2007**, *13*, 725–742.
17. deFur, P.L.; Evans, G.W.; Hubal, E.A.C.; Kyle, A.D.; Morello-Frosch, R.A.; Williams, D.R. Vulnerability as a function of individual and group resources in cumulative risk assessment. *Environ. Health Perspect.* **2007**, *115*, 817–824.
18. Hennig, B.; Ormsbee, L.; McClain, C.J.; Watkins, B.A.; Blumberg, B.; Bachas, L.G.; Sanderson, W.; Thompson, C.; Suk, W.A. Nutrition can modulate the toxicity of environmental pollutants: Implications in risk assessment and human health. *Environ. Health Perspect.* **2012**, *120*, 771–774.
19. Howd, R.A. Considering changes in exposure and sensitivity in an early life cumulative risk assessment. *Int. J. Toxicol.* **2009**, *29*, 71–77.
20. Knol, A.B.; Briggs, D.J.; Lebret, E. Assessment of complex environmental health problems: Framing the structures and structuring the frameworks. *Sci. Total Enviro.* **2010**, *408*, 2785–2794.
21. Lentz, T.J.; Dotson, G.S.; Williams, P.R.D.; Maier, A.; Gadagbui, B.; Pandalai, S.P.; Lamba, A.; Hearl, F.; Mumtaz, M. Aggregate exposure and cumulative risk assessment—Integrating occupational and non-occupational risk factors. *J. Occup. Environ. Hyg.* **2015**, *12*, S112–S126.
22. Levy, J.I. Is epidemiology the key to cumulative risk assessment? *Risk Anal.* **2008**, *28*, 1507–1513.
23. Linder, S.H.; Sexton, K. Conceptual models for cumulative risk assessment. *Am. J. Public Health* **2011**, *101*, S74–S81.
24. Menzie, C.A.; MacDonell, M.M.; Mumtaz, M. A phased approach for assessing combined effects from multiple stressors. *Environ. Health Perspect.* **2007**, *115*, 807–816.

25. Morello-Frosch, R.; Zuk, M.; Jerrett, M.; Shamasunder, B.; Kyle, A.D. Understanding the cumulative impacts of inequalities in environmental health: Implications for policy. *Health Aff.* **2011**, *30*, 879–887.
26. Segal, D.; Lin, Y.-S.; Ginsberg, G.; Sonawane, B. A conceptual framework for evaluating the interaction of a chemical and nonchemical stressor in human health risk assessments: A case study for lead and psychosocial stress. *Hum. Ecol. Risk Assess. Int. J.* **2015**, *21*, 1840–1868.
27. Sexton, K. Cumulative health risk assessment: Finding new ideas and escaping from the old ones. *Hum. Ecol. Risk Assess. Int. J.* **2014**, *21*, 934–951.
28. Sexton, K.; Linder, S.H. The role of cumulative risk assessment in decisions about environmental justice. *Int. J. Environ. Res. Public Health* **2010**, *7*, 4037–4049.
29. Smith, M.T.; de la Rosa, R.; Daniels, S.I. Using exposomics to assess cumulative risks and promote health. *Environ. Mol. Mutagen.* **2015**, *56*, 715–723.
30. Juarez, P.; Matthews-Juarez, P.; Hood, D.; Im, W.; Levine, R.; Kilbourne, B.; Langston, M.; Al-Hamdan, M.; Crosson, W.; Estes, M.; et al. The public health exposome: A population-based, exposure science approach to health disparities research. *Int. J. Environ. Res. Public Health* **2014**, *11*, 12866–12895.
31. Rider, C.V.; Dourson, M.L.; Hertzberg, R.C.; Mumtaz, M.M.; Price, P.S.; Simmons, J.E. Incorporating nonchemical stressors into cumulative risk assessments. *Toxicol. Sci.* **2012**, *127*, 10–17.
32. Rider, C.V.; Boekelheide, K.; Catlin, N.; Gordon, C.J.; Morata, T.; Selgrade, M.K.; Sexton, K.; Simmons, J.E. Cumulative risk: Toxicity and interactions of physical and chemical stressors. *Toxicol. Sci.* **2013**, *137*, 3–11.
33. Ryan, P.B.; Burke, T.A.; Cohen Hubal, E.A.; Cura, J.J.; McKone, T.E. Using biomarkers to inform cumulative risk assessment. *Environ. Health Perspect.* **2007**, *115*, 833–840.
34. Sexton, K.; Linder, S.H. Cumulative risk assessment for combined health effects from chemical and nonchemical stressors. *Am. J. Public Health* **2011**, *101*, S81–S88.
35. Su, J.G.; Morello-Frosch, R.; Jesdale, B.M.; Kyle, A.D.; Shamasunder, B.; Jerrett, M. An index for assessing demographic inequalities in cumulative environmental hazards with application to Los Angeles, California. *Environ. Sci. Technol.* **2009**, *43*, 7626–7634.
36. Zeise, L.; Bois, F.Y.; Chiu, W.A.; Hattis, D.; Rusyn, I.; Guyton, K.Z. Addressing human variability in next-generation human health risk assessments of environmental chemicals. *Environ. Health Perspect.* **2013**, *121*, 23–31.
37. Aagaard-Hansen, J.; Sørensen, B.H.; Chaignat, C.L. A comprehensive approach to risk assessment and surveillance guiding public health interventions. *Trop. Med. Int. Health* **2009**, *14*, 1034–1039.
38. Basara, H.G.; Yuan, M. Community health assessment using self-organizing maps and geographic information systems. *Int. J. Health Geogr.* **2008**, *7*, 67.
39. Andrey, J.; Jones, B. The dynamic nature of social disadvantage: Implications for hazard exposure and vulnerability in Greater Vancouver. *Can. Geogr.* **2008**, *52*, 146–168.
40. Briggs, D.; Abellan, J.J.; Fecht, D. Environmental inequity in England: Small area associations between socio-economic status and environmental pollution. *Soc. Sci. Med.* **2008**, *67*, 1612–1629.
41. Huang, G.; London, J.K. Cumulative environmental vulnerability and environmental justice in California's San Joaquin Valley. *Int. J. Environ. Res. Public Health* **2012**, *9*, 1593–1608.
42. MacLachlan, J.C.; Jerrett, M.; Abernathy, T.; Sears, M.; Bunch, M.J. Mapping health on the internet: A new tool for environmental justice and public health research. *Health Place* **2007**, *13*, 72–86.
43. Salinas, J.J.; Shah, M.; Abdelbary, B.; Gay, J.L.; Sexton, K. Application of a novel method for assessing cumulative risk burden by county. *Int. J. Environ. Res. Public Health* **2012**, *9*, 1820–1835.
44. Shmool, J.L.C.; Kubzansky, L.D.; Dotson Newman, O.; Spengler, J.; Shepard, P.; Clougherty, J.E. Social stressors and air pollution across New York city communities: A spatial approach for assessing correlations among multiple exposures. *Environ. Health* **2014**, *13*, doi:10.1186/1476-069x-13-91.
45. Tornero-Velez, R.; Egeghy, P.P.; Cohen Hubal, E.A. Biogeographical analysis of chemical co-occurrence data to identify priorities for mixtures research. *Risk Anal.* **2011**, *32*, 224–236.
46. Wang, B.; Chen, Z. A GIS-based fuzzy aggregation modeling approach for air pollution risk assessment. In *2010 Seventh International Conference on Fuzzy Systems and Knowledge Discovery*; Institute of Electrical and Electronics Engineers (IEEE): New York, NY, USA, 2010.
47. Zartarian, V.G.; Schultz, B.D.; Barzyk, T.M.; Smuts, M.; Hammond, D.M.; Medina-Vera, M.; Geller, A.M. The environmental protection agency's community-focused exposure and risk screening tool (C-FERST) and its potential use for environmental justice efforts. *Am. J. Public Health* **2011**, *101*, S286–S294.

48. Al Zabadi, H.; Ferrari, L.; Sari-Minodier, I.; Kerautret, M.-A.; Tiberguent, A.; Paris, C.; Zmirou-Navier, D. Integrated exposure assessment of sewage workers to genotoxicants: An urinary biomarker approach and oxidative stress evaluation. *Environ. Health* **2011**, *10*, doi:10.1186/1476-069X-10-23.
49. Das, P.; Shaik, A.; Jamil, K. Genotoxicity induced by pesticide mixtures: In-vitro studies on human peripheral blood lymphocytes. *Toxicol. Ind. Health* **2007**, *23*, 449–458.
50. Daughton, C.G. Using biomarkers in sewage to monitor community-wide human health: Isoprostanes as conceptual prototype. *Sci. Total Environ.* **2012**, *424*, 16–38.
51. Gennings, C.; Ellis, R.; Ritter, J.K. Linking empirical estimates of body burden of environmental chemicals and wellness using NHANES data. *Environ. Int.* **2012**, *39*, 56–65.
52. Whyatt, R.M.; Liu, X.; Rauh, V.A.; Calafat, A.M.; Just, A.C.; Hoepner, L.; Diaz, D.; Quinn, J.; Adibi, J.; Perera, F.P.; et al. Maternal prenatal urinary phthalate metabolite concentrations and child mental, psychomotor, and behavioral development at 3 years of age. *Environ. Health Perspect.* **2011**, *120*, 290–295.
53. Bonefeld-Jørgensen, E.C.; Ghisari, M.; Wielsøe, M.; Bjerregaard-Olesen, C.; Kjeldsen, L.S.; Long, M. Biomonitoring and hormone-disrupting effect biomarkers of persistent organic pollutantsin vitroandex vivo. *Basic Clin. Pharmacol. Toxicol.* **2014**, *115*, 118–128.
54. Hendriksen, P.J.M.; Freidig, A.P.; Jonker, D.; Thissen, U.; Bogaards, J.J.P.; Mumtaz, M.M.; Groten, J.P.; Stierum, R.H. Transcriptomics analysis of interactive effects of benzene, trichloroethylene and methyl mercury within binary and ternary mixtures on the liver and kidney following subchronic exposure in the rat. *Toxicol. Appl. Pharmacol.* **2007**, *225*, 171–188.
55. Miyake, K.; Yang, W.; Hara, K.; Yasuda, K.; Horikawa, Y.; Osawa, H.; Furuta, H.; Ng, M.C.Y.; Hirota, Y.; Mori, H.; et al. Construction of a prediction model for type 2 diabetes mellitus in the Japanese population based on 11 genes with strong evidence of the association. *J. Hum. Genet.* **2009**, *54*, 236–241.
56. North, K.E.; Martin, L.J. The importance of gene-environment interaction: Implications for social scientists. *Sociol. Methods Res.* **2008**, *37*, 164–200.
57. Thayer, Z.M.; Kuzawa, C.W. Biological memories of past environments: Epigenetic pathways to health disparities. *Epigenetics* **2011**, *6*, 798–803.
58. Barzyk, T.M.; Conlon, K.C.; Chahine, T.; Hammond, D.M.; Zartarian, V.G.; Schultz, B.D. Tools available to communities for conducting cumulative exposure and risk assessments. *J. Expo. Sci. Environ. Epidemiol.* **2010**, *20*, 371–384.
59. Borgert, C.J.; Sargent, E.V.; Casella, G.; Dietrich, D.R.; McCarty, L.S.; Golden, R.J. The human relevant potency threshold: Reducing uncertainty by human calibration of cumulative risk assessments. *Regul. Toxicol. Pharmacol.* **2012**, *62*, 313–328.
60. Boyd, J.; Vrana, J.A.; Williams, H.N. In vitro approach to predict post-translational phosphorylation response to mixtures. *Toxicology* **2013**, *313*, 113–121.
61. Chen, D.G. A quantal statistical isobologram model to identify joint action for chemical mixtures. *Environmetrics* **2009**, *20*, 101–109.
62. Clougherty, J.E.; Rossi, C.A.; Lawrence, J.; Long, M.S.; Diaz, E.A.; Lim, R.H.; McEwen, B.; Koutrakis, P.; Godleski, J.J. Chronic social stress and susceptibility to concentrated ambient fine particles in rats. *Environ. Health Perspect.* **2010**, *118*, 769–775.
63. Evans, G.W.; Becker, F.D.; Zahn, A.; Bilotta, E.; Keesee, A.M. Capturing the ecology of workplace stress with cumulative risk assessment. *Environ. Behav.* **2010**, *44*, 136–154.
64. Glass, T.A.; Bandeen-Roche, K.; McAtee, M.; Bolla, K.; Todd, A.C.; Schwartz, B.S. Neighborhood psychosocial hazards and the association of cumulative lead dose with cognitive function in older adults. *Am. J. Epidemiol.* **2009**, *169*, 683–692.
65. Glei, D.A.; Goldman, N.; Chuang, Y.-L.; Weinstein, M. Do chronic stressors lead to physiological dysregulation? Testing the theory of allostatic load. *Psychosom. Med.* **2007**, *69*, 769–776.
66. Hertzberg, R.C.; Pan, Y.; Li, R.; Haber, L.T.; Lyles, R.H.; Herr, D.W.; Moser, V.C.; Simmons, J.E. Corrigendum to a four-step approach to evaluate mixtures for consistency with dose addition (toxicology 313 (November 2013) 134–144). *Toxicology* **2015**, *337*, 108.
67. MacDonell, M.M.; Haroun, L.A.; Teuschler, L.K.; Rice, G.E.; Hertzberg, R.C.; Butler, J.P.; Chang, Y.-S.; Clark, S.L.; Johns, A.P.; Perry, C.S.; et al. Cumulative risk assessment toolbox: Methods and approaches for the practitioner. *J. Toxicol.* **2013**, *2013*, 1–36.

68. Marshall, S.; Gennings, C.; Teuschler, L.K.; Stork, L.G.; Tornero-Velez, R.; Crofton, K.M.; Rice, G.E. An empirical approach to sufficient similarity: Combining exposure data and mixtures toxicology data. *Risk Anal.* **2013**, *33*, 1582–1595.
69. Medina-Vera, M.; Van Emon, J.M.; Melnyk, L.J.; Bradham, K.D.; Harper, S.L.; Morgan, J.N. An overview of measurement method tools available to communities for conducting exposure and cumulative risk assessments. *J. Expo. Sci. Environ. Epidemiol.* **2009**, *20*, 359–370.
70. Navas-Acien, A.; Tellez-Plaza, M.; Guallar, E.; Muntner, P.; Silbergeld, E.; Jaar, B.; Weaver, V. Blood cadmium and lead and chronic kidney disease in U.S. adults: A joint analysis. *Am. J. Epidemiol.* **2009**, *170*, 1156–1164.
71. Ragas, A.M.J.; Oldenkamp, R.; Preeker, N.L.; Wernicke, J.; Schlink, U. Cumulative risk assessment of chemical exposures in urban environments. *Environ. Int.* **2011**, *37*, 872–881.
72. Scholze, M.; Silva, E.; Kortenkamp, A. Extending the applicability of the dose addition model to the assessment of chemical mixtures of partial agonists by using a novel toxic unit extrapolation method. *PLoS ONE* **2014**, *9*, e88808.
73. Silva, E.; Rajapakse, N.; Scholze, M.; Backhaus, T.; Ermler, S.; Kortenkamp, A. Joint effects of heterogeneous estrogenic chemicals in the e-screen—Exploring the applicability of concentration addition. *Toxicol. Sci.* **2011**, *122*, 383–394.
74. Zota, A.R.; Shenassa, E.D.; Morello-Frosch, R. Allostatic load amplifies the effect of blood lead levels on elevated blood pressure among middle-aged U.S. adults: A cross-sectional study. *Environ. Health* **2013**, *12*, doi:10.1186/1476-069X-12-64.
75. Kondo, M.C.; Gross-Davis, C.A.; May, K.; Davis, L.O.; Johnson, T.; Mallard, M.; Gabbadon, A.; Sherrod, C.; Branas, C.C. Place-based stressors associated with industry and air pollution. *Health Place* **2014**, *28*, 31–37.
76. Bobb, J.F.; Valeri, L.; Claus Henn, B.; Christiani, D.C.; Wright, R.O.; Mazumdar, M.; Godleski, J.J.; Coull, B.A. Bayesian kernel machine regression for estimating the health effects of multi-pollutant mixtures. *Biostatistics* **2014**, *16*, 493–508.
77. Tan, Y.-M.; Clewell, H.; Campbell, J.; Andersen, M. Evaluating pharmacokinetic and pharmacodynamic interactions with computational models in supporting cumulative risk assessment. *Int. J. Environ. Res. Public Health* **2011**, *8*, 1613–1630.
78. Tie, Y.; McPhail, B.; Hong, H.; Pearce, B.A.; Schnackenberg, L.K.; Ge, W.; Buzatu, D.A.; Wilkes, J.G.; Fuscoe, J.C.; Tong, W.; et al. Modeling chemical interaction profiles: II. Molecular docking, spectral data-activity relationship, and structure-activity relationship models for potent and weak inhibitors of cytochrome p450 cyp3a4 isozyme. *Molecules* **2012**, *17*, 3407–3460.
79. Wason, S.C.; Smith, T.J.; Perry, M.J.; Levy, J.I. Using physiologically-based pharmacokinetic models to incorporate chemical and non-chemical stressors into cumulative risk assessment: A case study of pesticide exposures. *Int. J. Environ. Res. Public Health* **2012**, *9*, 1971–1983.
80. Levy, J.I.; Fabian, M.P.; Peters, J.L. Meta-Analytic approaches for multistressor dose-response function development: Strengths, limitations, and case studies. *Risk Anal.* **2014**, *35*, 1040–1049.
81. Evans, A.; Rice, G.; Teuschler, L.; Wright, J. Joint exposure to chemical and nonchemical neurodevelopmental stressors in U.S. women of reproductive age in NHANES. *Int. J. Environ. Res. Public Health* **2014**, *11*, 4384–4401.
82. Alexeeff, G.V.; Faust, J.B.; August, L.M.; Milanes, C.; Randles, K.; Zeise, L.; Denton, J. A screening method for assessing cumulative impacts. *Int. J. Environ. Res. Public Health* **2012**, *9*, 648–659.
83. Bevc, C.A.; Marshall, B.K.; Picou, J.S. Environmental justice and toxic exposure: Toward a spatial model of physical health and psychological well-being. *Soc. Sci. Res.* **2007**, *36*, 48–67.
84. Chen, E.; Schreier, H.M.C.; Strunk, R.C.; Brauer, M. Chronic traffic-related air pollution and stress interact to predict biologic and clinical outcomes in asthma. *Environ. Health Perspect.* **2008**, *116*, 970–975.
85. Dulin-Keita, A.; Casazza, K.; Fernandez, J.R.; Goran, M.I.; Gower, B. Do neighbourhoods matter? Neighbourhood disorder and long-term trends in serum cortisol levels. *J. Epidemiol. Community Health* **2010**, *66*, 24–29.
86. Hicken, M.T.; Gee, G.C.; Connell, C.; Snow, R.C.; Morenoff, J.; Hu, H. Black-white blood pressure disparities: Depressive symptoms and differential vulnerability to blood lead. *Environ. Health Perspect.* **2013**, *121*, 205–209.

87. Hoffmann, B.; Kolahgar, B.; Rauchfuss, K.; Eberwein, G.; Franzen-Reuter, I.; Kraft, M.; Wilhelm, M.; Ranft, U.; Jöckel, K.-H. Childhood social position and associations between environmental exposures and health outcomes. *Int. J. Hyg. Environ. Health* **2009**, *212*, 146–156.
88. Islam, T.; Urman, R.; Gauderman, W.J.; Milam, J.; Lurmann, F.; Shankardass, K.; Avol, E.; Gilliland, F.; McConnell, R. Parental stress increases the detrimental effect of traffic exposure on children's lung function. *Am. J. Respir. Crit. Care Med.* **2011**, *184*, 822–827.
89. Pearlman, D.N. Neighborhood-Level risk and resilience factors: An emerging issue in childhood asthma epidemiology. *Expert Rev. Clin. Immunol.* **2009**, *5*, 633–637.
90. Wright, R.J.; Suglia, S.F.; Levy, J.; Fortun, K.; Shields, A.; Subramanian, S.V.; Wright, R. Transdisciplinary research strategies for understanding socially patterned disease: The asthma coalition on community, environment, and social stress (access) project as a case study. *Ciência Saúde Coletiva* **2008**, *13*, 1729–1742.
91. Schulz, A.J.; Mentz, G.; Lachance, L.; Johnson, J.; Gaines, C.; Israel, B.A. Associations between socioeconomic status and allostatic load: Effects of neighborhood poverty and tests of mediating pathways. *Am. J. Public Health* **2012**, *102*, 1706–1714.
92. Shankardass, K.; McConnell, R.; Jerrett, M.; Milam, J.; Richardson, J.; Berhane, K. Parental stress increases the effect of traffic-related air pollution on childhood asthma incidence. *Proc. Natl. Acad. Sci. USA* **2009**, *106*, 12406–12411.
93. Theall, K.P.; Brett, Z.H.; Shirtcliff, E.A.; Dunn, E.C.; Drury, S.S. Neighborhood disorder and telomeres: Connecting children's exposure to community level stress and cellular response. *Soc. Sci. Med.* **2013**, *85*, 50–58.
94. Erickson, A.C.; Arbour, L. The shared pathoetiological effects of particulate air pollution and the social environment on fetal-placental development. *J. Environ. Public Health* **2014**, *2014*, 1–20.
95. Wing, S.; Horton, R.A.; Muhammad, N.; Grant, G.R.; Tajik, M.; Thu, K. Integrating epidemiology, education, and organizing for environmental justice: Community health effects of industrial hog operations. *Am. J. Public Health* **2008**, *98*, 1390–1397.
96. Horton, R.A.; Wing, S.; Marshall, S.W.; Brownley, K.A. Malodor as a trigger of stress and negative mood in neighbors of industrial hog operations. *Am. J. Public Health* **2009**, *99*, S610–S615.
97. Schinasi, L.; Horton, R.A.; Guidry, V.T.; Wing, S.; Marshall, S.W.; Morland, K.B. Air pollution, lung function, and physical symptoms in communities near concentrated swine feeding operations. *Epidemiology* **2011**, *22*, 208–215.
98. Wing, S.; Horton, R.A.; Rose, K.M. Air pollution from industrial swine operations and blood pressure of neighboring residents. *Environ. Health Perspect.* **2013**, *121*, 92–96.
99. Young, G.S.; Fox, M.A.; Trush, M.; Kanarek, N.; Glass, T.A.; Curriero, F.C. Differential exposure to hazardous air pollution in the United States: A multilevel analysis of urbanization and neighborhood socioeconomic deprivation. *Int. J. Environ. Res. Public Health* **2012**, *9*, 2204–2225.
100. Schüle, S.A.; Bolte, G. Interactive and independent associations between the socioeconomic and objective built environment on the neighbourhood level and individual health: A systematic review of multilevel studies. *PLoS ONE* **2015**, *10*, e0123456.
101. McDonald, Y.J.; Grineski, S.E.; Collins, T.W.; Kim, Y.-A. A scalable climate health justice assessment model. *Soc. Sci. Med.* **2015**, *133*, 242–252.
102. Holmstrup, M.; Bindesbøl, A.-M.; Oostingh, G.J.; Duschl, A.; Scheil, V.; Köhler, H.-R.; Loureiro, S.; Soares, A.M.V.M.; Ferreira, A.L.G.; Kienle, C.; et al. Interactions between effects of environmental chemicals and natural stressors: A review. *Sci. Total Environ.* **2010**, *408*, 3746–3762.
103. Jansen, M.; Coors, A.; Stoks, R.; De Meester, L. Evolutionary ecotoxicology of pesticide resistance: A case study in Daphnia. *Ecotoxicology* **2011**, *20*, 543–551.
104. Løkke, H. Novel methods for integrated risk assessment of cumulative stressors—Results from the NOMIRACLE project. *Sci. Total Environ.* **2010**, *408*, 3719–3724.
105. Moe, S.J.; De Schamphelaere, K.; Clements, W.H.; Sorensen, M.T.; Van den Brink, P.J.; Liess, M. Combined and interactive effects of global climate change and toxicants on populations and communities. *Environ. Toxicol. Chem.* **2012**, *32*, 49–61.
106. Al-Salhi, R.; Abdul-Sada, A.; Lange, A.; Tyler, C.R.; Hill, E.M. The xenometabolome and novel contaminant markers in fish exposed to a wastewater treatment works effluent. *Environ. Sci. Technol.* **2012**, *46*, 9080–9088.

107. Baylay, A.J.; Spurgeon, D.J.; Svendsen, C.; Griffin, J.L.; Swain, S.C.; Sturzenbaum, S.R.; Jones, O.A.H. A metabolomics based test of independent action and concentration addition using the earthworm *Lumbricus rubellus*. *Ecotoxicology* **2012**, *21*, 1436–1447.
108. Dondero, F.; Negri, A.; Boatti, L.; Marsano, F.; Mignone, F.; Viarengo, A. Transcriptomic and proteomic effects of a neonicotinoid insecticide mixture in the marine mussel (*Mytilus galloprovincialis*, Lam.). *Sci. Total Environ.* **2010**, *408*, 3775–3786.
109. Garcia-Reyero, N.; Escalon, B.L.; Loh, P.R.; Laird, J.G.; Kennedy, A.J.; Berger, B.; Perkins, E.J. Assessment of chemical mixtures and groundwater effects on *Daphnia magna* transcriptomics. *Environ. Sci. Technol.* **2012**, *46*, 42–50.
110. Wang, Z.; Chen, J.; Huang, L.; Wang, Y.; Cai, X.; Qiao, X.; Dong, Y. Integrated fuzzy concentration addition-independent action (IFCA-IA) model outperforms two-stage prediction (TSP) for predicting mixture toxicity. *Chemosphere* **2009**, *74*, 735–740.
111. Zhang, Y.-H.; Liu, S.-S.; Liu, H.-L.; Liu, Z.-Z. Evaluation of the combined toxicity of 15 pesticides by uniform design. *Pest Manag. Sci.* **2010**, *66*, 879–887.
112. Zou, X.; Zhou, X.; Lin, Z.; Deng, Z.; Yin, D. A docking-based receptor library of antibiotics and its novel application in predicting chronic mixture toxicity for environmental risk assessment. *Environ. Monit. Assess.* **2012**, *185*, 4513–4527.
113. Landis, W.G.; Ayre, K.K.; Johns, A.F.; Summers, H.M.; Stinson, J.; Harris, M.J.; Herring, C.E.; Markiewicz, A.J. The multiple stressor ecological risk assessment for the mercury contaminated South River and Upper Shenandoah River using the Bayesian network-relative risk model. *Integr. Environ. Assess. Manag.* **2016**, doi:10.1002/ieam.1758.
114. Dietrich, J.P.; Van Gaest, A.L.; Strickland, S.A.; Arkoosh, M.R. The impact of temperature stress and pesticide exposure on mortality and disease susceptibility of endangered pacific salmon. *Chemosphere* **2014**, *108*, 353–359.
115. Langmead, O.; McQuatters-Gollop, A.; Mee, L.D.; Friedrich, J.; Gilbert, A.J.; Gomoiu, M.-T.; Jackson, E.L.; Knudsen, S.; Minicheva, G.; Todorova, V. Recovery or decline of the northwestern black sea: A societal choice revealed by socio-ecological modelling. *Ecol. Modell.* **2009**, *220*, 2927–2939.
116. McConnachie, S.H.; O'Connor, C.M.; Gilmour, K.M.; Iwama, G.K.; Cooke, S.J. Supraphysiological cortisol elevation alters the response of wild bluegill sunfish to subsequent stressors. *J. Exp. Zool. Part A: Ecol. Genet. Physiol.* **2012**, *317*, 321–332.
117. Stampfli, N.C.; Knillmann, S.; Liess, M.; Noskov, Y.A.; Schäfer, R.B.; Beketov, M.A. Two stressors and a community—Effects of hydrological disturbance and a toxicant on freshwater zooplankton. *Aquat. Toxicol.* **2013**, *127*, 9–20.
118. Vanhoudt, N.; Vandenhove, H.; Real, A.; Bradshaw, C.; Stark, K. A review of multiple stressor studies that include ionising radiation. *Environ. Pollut.* **2012**, *168*, 177–192.
119. Vidau, C.; Diogon, M.; Aufauvre, J.; Fontbonne, R.; Viguès, B.; Brunet, J.-L.; Texier, C.; Biron, D.G.; Blot, N.; El Alaoui, H.; et al. Exposure to sublethal doses of fipronil and thiacloprid highly increases mortality of honeybees previously infected by *Nosema ceranae*. *PLoS ONE* **2011**, *6*, e21550.
120. Vieira, L.R.; Guilhermino, L. Multiple stress effects on marine planktonic organisms: Influence of temperature on the toxicity of polycyclic aromatic hydrocarbons to *Tetraselmis chuii*. *J. Sea Res.* **2012**, *72*, 94–98.
121. De Laender, F.; Janssen, C.R. Brief communication: The ecosystem perspective in ecotoxicology as a way forward for the ecological risk assessment of chemicals. *Integr. Environ. Assess. Manag.* **2013**, *9*, e34–e38.
122. Myers, S.S.; Gaffikin, L.; Golden, C.D.; Ostfeld, R.S.; H. Redford, K.; H. Ricketts, T.; Turner, W.R.; Osofsky, S.A. Human health impacts of ecosystem alteration. *Proc. Natl. Acad. Sci. USA* **2013**, *110*, 18753–18760.
123. Reis, S.; Morris, G.; Fleming, L.E.; Beck, S.; Taylor, T.; White, M.; Depledge, M.H.; Steinle, S.; Sabel, C.E.; Cowie, H.; et al. Integrating health and environmental impact analysis. *Public Health* **2015**, *129*, 1383–1389.
124. Jackson, L.E.; Daniel, J.; McCorkle, B.; Sears, A.; Bush, K.F. Linking ecosystem services and human health: The eco-health relationship browser. *Int. J. Public Health* **2013**, *58*, 747–755.
125. Norman, L.M.; Villarreal, M.L.; Lara-Valencia, F.; Yuan, Y.; Nie, W.; Wilson, S.; Amaya, G.; Sleeter, R. Mapping socio-environmentally vulnerable populations access and exposure to ecosystem services at the U.S.-Mexico borderlands. *Appl. Geogr.* **2012**, *34*, 413–424.

126. Ringold, P.L.; Boyd, J.; Landers, D.; Weber, M. What data should we collect? A framework for identifying indicators of ecosystem contributions to human well-being. *Front. Ecol. Environ.* **2013**, *11*, 98–105.
127. Smith, L.M.; Case, J.L.; Smith, H.M.; Harwell, L.C.; Summers, J.K. Relating ecosystem services to domains of human well-being: Foundation for a U.S. index. *Ecol. Indic.* **2013**, *28*, 79–90.
128. Yang, W.; Dietz, T.; Liu, W.; Luo, J.; Liu, J. Going beyond the millennium ecosystem assessment: An index system of human dependence on ecosystem services. *PLoS ONE* **2013**, *8*, e64581.

© 2017 by the authors; licensee MDPI, Basel, Switzerland. This article is an open access article distributed under the terms and conditions of the Creative Commons by Attribution (CC-BY) license (http://creativecommons.org/licenses/by/4.0/).
